# RNA modification: mechanisms and therapeutic targets

**DOI:** 10.1186/s43556-023-00139-x

**Published:** 2023-08-24

**Authors:** Lei Qiu, Qian Jing, Yanbo Li, Junhong Han

**Affiliations:** 1 State Key Laboratory of Biotherapy and Cancer Center, Research Laboratory of Tumor Epigenetics and Genomics, Frontiers Science Center for Disease-Related Molecular Network, West China Hospital, Sichuan University, Chengdu, 610041 P.R. China; 2 Department of Neurology, West China Hospital, Sichuan University, Chengdu, 610041 China

**Keywords:** RNA modification, Cancer, Neurological disorders, Cardiovascular diseases, Metabolic diseases, Inhibitors

## Abstract

RNA modifications are dynamic and reversible chemical modifications on substrate RNA that are regulated by specific modifying enzymes. They play important roles in the regulation of many biological processes in various diseases, such as the development of cancer and other diseases. With the help of advanced sequencing technologies, the role of RNA modifications has caught increasing attention in human diseases in scientific research. In this review, we briefly summarized the basic mechanisms of several common RNA modifications, including m6A, m5C, m1A, m7G, Ψ, A-to-I editing and ac4C. Importantly, we discussed their potential functions in human diseases, including cancer, neurological disorders, cardiovascular diseases, metabolic diseases, genetic and developmental diseases, as well as immune disorders. Through the “writing-erasing-reading” mechanisms, RNA modifications regulate the stability, translation, and localization of pivotal disease-related mRNAs to manipulate disease development. Moreover, we also highlighted in this review all currently available RNA-modifier-targeting small molecular inhibitors or activators, most of which are designed against m6A-related enzymes, such as METTL3, FTO and ALKBH5. This review provides clues for potential clinical therapy as well as future study directions in the RNA modification field. More in-depth studies on RNA modifications, their roles in human diseases and further development of their inhibitors or activators are needed for a thorough understanding of epitranscriptomics as well as diagnosis, treatment, and prognosis of human diseases.

## Introduction

RNA molecules like DNA and proteins are also chemically modified through RNA modifying enzymes. With the fast development of detecting technologies, such as chemical labeling, mass spectrometry and high-throughput sequencing, more than 170 different types of post-transcriptional modifications on RNA have been identified, dynamically regulating RNA functions and stability [1–5]. RNA modifications target all four RNA bases and the ribose sugar, as well as all known RNA species [6]. RNA modifications play critical roles in a variety of cellular processes, especially in the regulation of mRNA stability and translation. For example, certain RNA modifications promote mRNA stability and enhance its translation into protein, while others may target mRNA for degradation and prevent its translation. RNA modifications are also involved in mRNA localization and alternative splicing.

RNA modifying enzymes can be classified as “writers”, “erasers” and “readers”. “Writers” are usually modifying enzyme complexes that install RNA modifications on RNA substrates [7]. Different types of “writers” have different preferences for installation sites; for example, Methyltransferase 3/14 (METTL3/14) complex preferentially installs m6A methylation in a sequence motif RRACH, whereas METTL16 prefers a UAC (m6A) GAGAA sequence in the bulge of a stem-loop structured RNA [8]. “Erasers” alter the modification level by removing the chemical marks installed by “writers”, thus they are generally de-modifying enzymes [7]. Diverse regulatory machinery can be recruited by binding proteins (“readers”) that recognize RNA modification marks on target RNAs to impact their fate [7].

Recent studies have revealed the important roles of RNA modifying enzymes in human diseases, including cancer, neurological disorders, cardiovascular diseases, metabolic diseases, as well as developmental and genetic disorders. For example, the m5C methyltransferase NOP2/Sun RNA methyltransferase 2 (NSUN2) is overexpressed in breast cancer and is correlated with cancer development and progression [9]. In contrast, the tRNA methyltransferase TRM9L is down-regulated in breast cancer cells and other cancers [10]. Several RNA methyltransferases have been linked to intellectual disability, such as the FtsJ RNA 2’-O-Methyltransferase 1 (FTSJ1) [11], the TRNA Methyltransferase 1 (TRMT1) [12, 13] and NSUN2 [14, 15]. Defects in A-to-I editing have also been linked to neurological diseases, such as amyotrophic lateral sclerosis (ALS), the most common adult-onset motor neuron disease [16, 17]. Numerous genetic birth defects and developmental defects involve mutations in RNA modifying enzymes, such as Cri du chat syndrome (NSUN1) [18], Dubowitz syndrome (NSUN2) [19], William-Beuren syndrome (Williams-Beuren syndrome chromosome region 22/22, WBSCR20/22, and NSUN5) [20], and Hutchinson-Gilford progeria syndrome (N-Acetyltransferase 10, NAT10) [21, 22]. RNA modification also plays a role in metabolic disorders as well as cardiovascular disease. Variation of the fat mass and obesity-associated protein (FTO) is associated with obesity and low concentration of leptin [23, 24]. METTL3-mediated m6A methylation is essential for a normal cardiomyocyte hypertrophic response [25]. METTL3 and AlkB Homolog 5 (ALKBH5) oppositely regulate m6A modification of the master regulator of lysosomal biogenesis and autophagy genes, Transcription Factor EB (TFEB), which dictates the fate of hypoxia/reoxygenation-treated cardiomyocytes [26].

The increasing understanding of RNA modification and its role in cellular processes has provided new potential in the diagnosis, treatment, and prevention of a variety of diseases. Therefore, in order to help researchers to thoroughly understand the roles of RNA modifications in diseases and ways to target these modifications for clinical purposes, we summarize in this review the functional mechanisms of seven of the better studied RNA modifications, including N6-methyladenosine, 5-methylcytosine, N1-methyladenosine, internal 7-methylguanosine, pseudouridine, adenosine-to-inosine editing, and N4-acetylcytidine. We also discuss currently available small molecules targeting these modification pathways as well as their applications in human diseases.

## Mechanisms of common RNA modifications

### N6-methyladenosine (m6A)

**Fig. 1:**
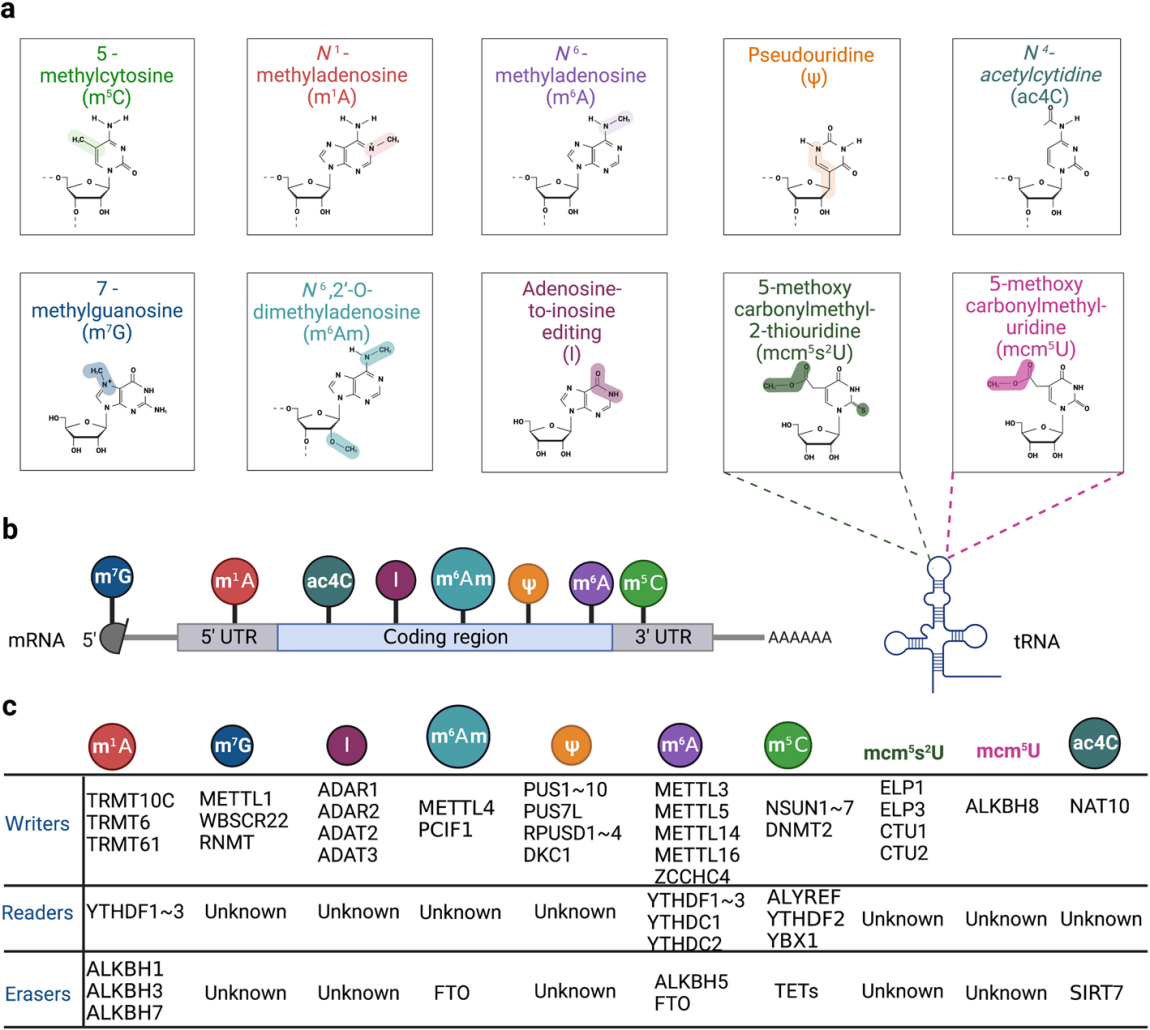
Eukaryotic RNA modifications. **a** The chemical structures of ten RNA modifications marking on ribose are presented. **b** Various RNA modifications are enriched in different regions of mRNA. m7G, m1A, m5C are enriched in 5’ cap, 5’ UTR, 3’ UTR regions, respectively. The other modifications are all enriched in CDS region. **c**. The various “writers”, “readers” and “erasers” associated with RNA modifications are listed in the table

To date, the methylation of internal adenosines at the N6 position (m6A) has been found in messenger RNA (mRNA) and non-coding RNAs (such as tRNA, rRNA, microRNA and long non-coding RNA) in eukaryotic cells (Fig. 1a-b) [27]. In the mid-1970s, m6A was first identified in mRNA as the most prominent chemical modification among more than 100 RNA modifications [28]. It accounts for approximately 50% of methylated ribonucleotides and affects over 7,000 mRNAs in individual transcriptomes of mammalian cells [28]. In the past several decades, accumulating evidence has demonstrated that m6A modification contributes to RNA fate decisions as well as functions such as mRNA stability, structural change, localization, transport, primary microRNA processing, translation and RNA–protein interactions [29]. Like DNA and histone methylations, m6A modification is a reversible and dynamic process relying on a variety of enzymes including “writers” (methyltransferases) [30–32], “erasers” (demethylases) [33–35], and “readers” (m6A-recognizing proteins) (Fig. 1c) [36–39]. This enzymatic system regulates the fate of target gene transcripts through addition, removal, and specific recognition of m6A modifications [40].

**Fig. 2:**
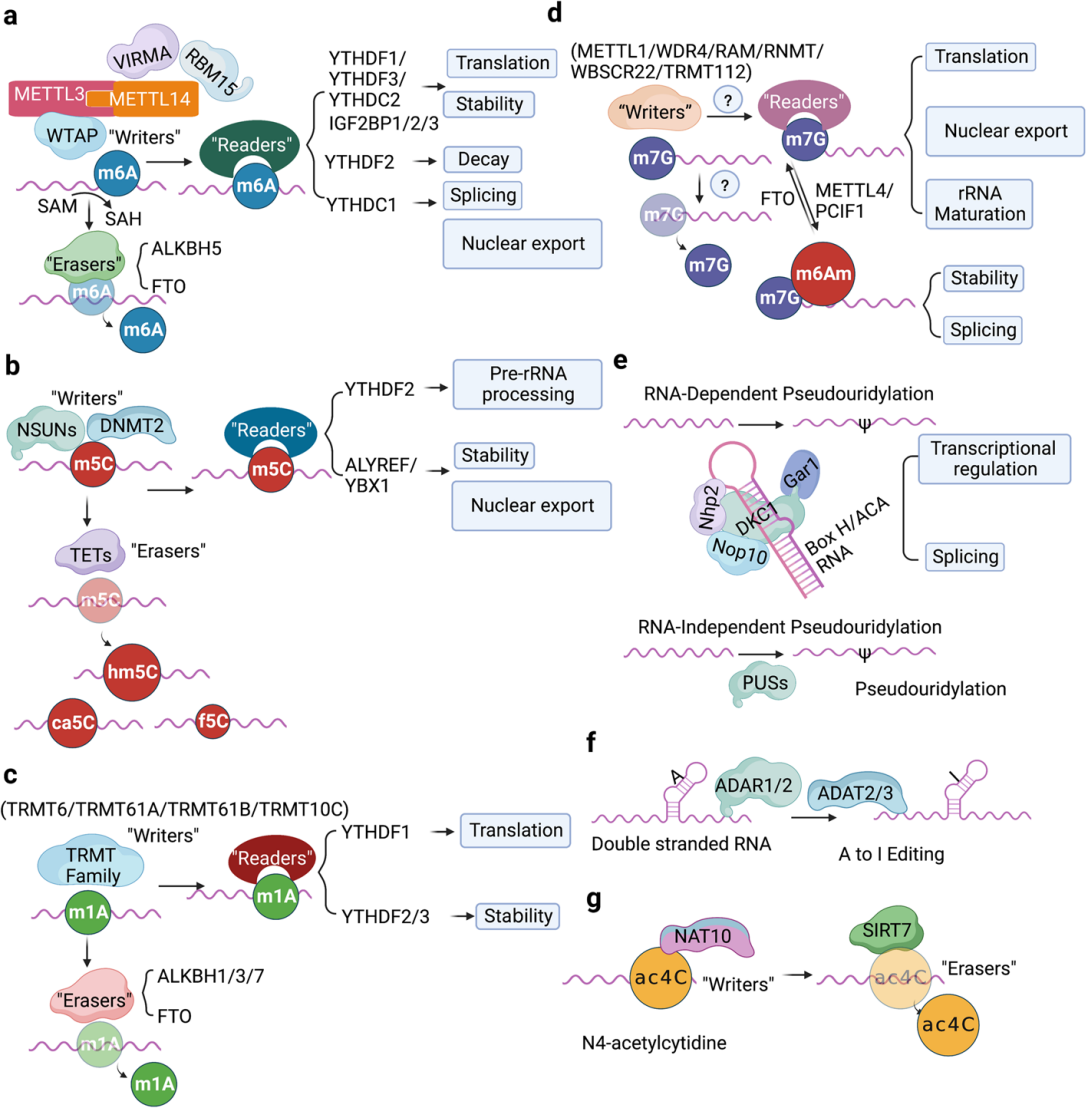
The molecular mechanisms of seven common RNA modifications. **a** The m6A methyltransferase complex components including METTL3-METTL14, VIRMA, RBM15, WTAP mediate m6A installation, whereas ALKBH5 and FTO function as “erasers” to remove m^6^A modification. YTHDF1 ~ 3, YTHDC1 ~ 2 and IGF2BP1 ~ 3 are responsible for “reading” m6A on substrate and lead to various phenotypical conditions, such as translation, enhanced RNA stability, RNA decay, RNA splicing or nuclear transport. **b** NSUNs and DNMT2 act as the m5C “writers” in mRNAs, while TET family enzymes can erase m5C by catalyzing the oxidative hydroxylation of m5C to hm5C, ca5C and f5C. YTHDF2, ALYREF and YBX1 recognize m5C and regulate the fate of substrates. **c** TRMT family proteins deposit m1A on substrate RNAs. m1A can be “read” by YTHDF1 ~ 3 or “erased” by ALKBH1/3/7 or FTO. **d** The m7G methyltransferase complex discovered currently includes METTL1/WDR4, WBSCR22/TRMT112, RNMT/RAM, whereas “erasers” or “readers” of m7G have not yet been reported. PCIF1/METTL4 add the m6Am modification adjacent to m7G; FTO can also remove m7G modification. **e** Pseudouridylation is mediated by either snoRNA-dependent or RNA-independent mechanism. DKC1 in combination with three core proteins (NOP10, GAR1 and NHP2) form the RNP complex, which is guided by box H/ACA snoRNAs to catalyze pseudouridylation; the PUS enzymes RNA-independently modify uridine to form pseudouridine. **f** ADAR1/2 and ADAT2/3 catalyze adenosine-to-inosine editing on double-stranded RNAs. **g** NAT10 is currently discovered the only one ac4C “writer”; SIRT7 is considered as a candidate “eraser”; the identity of the ac4C “readers” are still undetermined

The methyltransferase complex consists of METTL3/METTL14/Wilm’s-tumor-1-associated protein (WTAP) triplet as well as several co-factors including RNA-binding motif protein 15 (RBM15)/15B, VIRMA and KIAA1429 (Fig. 2a) [41, 42]. METTL3 containing active methyltransferase domain transfers methyl group from S-adenosylmethionine (SAM) to the adenosine (A) residue on the substrate [43, 44]. METTL14 supports METTL3 in recognizing RNA substrates as a critical component [45]. m6A modified site particularly localizes at the beginning of the 3′ untranslated region (UTR) near the stop codon, normally embedded within the consensus motif 5′-RRACH-3′ (R stands for G, A or U; H stands for U, A or C; Fig. 1b) [46–48]. The METTL3-METTL14 heterodimer binds to WTAP, which acts as an adaptor protein interacting with methyltransferases even though it has no catalytic methylation activity [49].

The dynamic regulation of m6A levels also involves two m6A demethylases, FTO and ALKBH5, which remove the methyl group from the adenosine on substrate (Fig. 2a) [50–52]. The presence of methyltransferases and demethylases together determines the level of m6A-"labeled" RNA [52].

The methylated RNA sites are recognized by “readers” [53]. Currently, YTH-domain containing proteins (YTHDs) were discovered as m6A “readers” that specifically bind m6A-modified RNA and regulate target RNA splicing, export, stability, decay and translation [54, 55]. In detail, YTHDF1, YTHDF3, YTHDC2 and Insulin like Growth Factor 2 mRNA Binding Protein 1/2/3 (IGF2BP1/2/3) are responsible for recruiting translation initiation factors to elevate RNA translation efficiency or influence RNA stability by modulating the rate of RNA degradation (Fig. 2a) [56, 57]. YTHDF family are m6A “readers” located in the cytoplasm including YTHDF1, YTHDF2, and YTHDF3 [58]. YTHDF1 exhibits the pro-translation effect in target mRNA by interacting with translation initiating factors and ribosomes (Fig. 2a) [59]. YTHDF2 recruits the CCR4-NOT deadenylase complex to facilitate the decay of target mRNAs (Fig. 2a) [60, 61]. YTHDF3 enhances both mRNA translation synergizing with YTHDF1 and mRNA decay mediated by YTHDF2 (Fig. 2a) [60, 62]. The other mammalian m6A “readers” with a YTH domain are YTHDC1 and YTHDC2 (Fig. 1c, 2a) [63]. YTHDC2 is also a cytoplasmic m6A reader that plays an essential role in RNA binding, mRNA translation, and degradation (Fig. 2a) [64, 65]. YTHDC1 is located in nucleus and guides RNA export from the nucleus by interacting with nuclear transport receptors (Fig. 2a) [66, 67]. Unlike YTH domain family proteins, IGF2BPs structurally contain four K homology (KH) domains and two RNA recognition motif (RRM) domains that stabilize mRNA instead of promoting mRNA degradation (Fig. 5d) [68]. IGF2BPs, including IGF2BP1/2/3, usually recognize a typical m6A motif-GG(m6A)C on target transcripts [69]. Recent studies reveal that IGF2BPs overexpress in various tumors and stabilize multiple RNAs such as SRY-box transcription factor 2 (SOX2), MYC, Transmembrane BAX Inhibitor Motif containing 6 (TMBIM6), and lncRNA HAGLR [12, 70].

m6A methylation is important for various physiological processes, including embryonic development, stress response, and cell fate determination [71].

Identification of writers, erasers and readers of m6A modification and development of m6A sequencing (m6A-seq) technologies have laid the foundation for studying the role of m6A mRNA modification in different diseases.

### N5-methylcytosine (m5C)

m5C was first discovered in 1925, known to occur on rRNA, tRNA, ncRNA and mRNA (Fig. 1a-b) [72, 73]. To date, 95,391 m5C sites have been uncovered in the human genome [74, 75]. Additionally, m5C is preferentially deposited in the proximity of the translation start sites, 3′-UTRs as well as near the Argonaute-binding regions in mRNAs (Fig. 1b) [76, 77]. m5C is written to target RNAs by DNA methyltransferase (DNMT) homologus DNMT2 and members of the NOL1/NOP2/SUN domain (NSUN) protein family, including seven members in eukaryotes (NSUN1-7, Fig. 1c, 2b) [78, 79]. NSUNs possess two catalytic cysteines in the active site, whereas DNMT2 utilizes only a single active site cysteine due to its DNA methyltransferase-like properties [80]. When m5C modification occurs on RNA, a covalent intermediate is formed between a cysteine in “writers” and the cytosine in target RNA, allowing carbon-5 to nucleophilic and attack SAM's methyl group [81].

5-methylcytosine in DNA (5mC) can be catalyzed by DNA dioxygenases ten-eleven translocations (TETs), including TET1/2/3, to its oxidized forms, 5-hydroxymethylcytosine (5hmC), 5-formylcytosine (5fC), and 5-carboxylcytosine (5caC) [13, 82]. Notably, TETs were found that its overexpression could significantly elevate the RNA hm5C level in recent research [83]. Moreover, TET1 was demonstrated to mediate oxidation of f5C to ca5C in RNA and TET2 is also involved in m5C oxidation in mRNA (Fig. 2b) [84, 85]. These discoveries prove that TETs also function as RNA demethylase to erase the m5C modification on substrate RNA. Meanwhile, ALKBH1, a dioxygenase for mitochondrial DNA and RNA, modulates mitochondrial activity by manipulating RNA m5C metabolism [86]. The m5C34 on mt-tRNA^Met^ and anticodon of cytoplasmic tRNA^Leu^ is oxidated to 5-hydroxymethyl-2’-O-methylcytidine (hm5Cm34) and 5-formyl-2’-O-methylcytidine (f5Cm34), suggesting that ALKBH1 mediates demethylation of m5C modification [87].

After the m5C modification of RNA, the m6A binding protein YTHDF2 also recognizes m5C-containing transcripts to facilitate pre-rRNA processing because it shares a conserved residue at the hydrophobic pocket that binds m5C-modified RNA (Fig. 1c, 2b) [88]. Recently, the Aly/REF export factor (ALYREF) has been identified as an mRNA m5C “reader” in the nucleus, promoting the nuclear export of m5C modified mRNAs (Fig. 1c, 2b) [89, 90]. Besides, DNA & RNA binding protein Y-box binding protein 1 (YBX1) can recognize and bind m5C modified mRNAs through its cold shock domain to stabilize mRNAs (Figs. 1c, 2b) [91]. Overall, the m5C regulatory network is complex and even more downstream effectors are yet to be discovered.

### N1-methyladenosine (m1A)

m1A, identified since the 1960s, is predominantly found in tRNA and rRNA (Fig. 1a) [92, 93]. Totally, the enrichment of m1A in mRNA is ten times less than that of m6A, only accounting for 0.05–0.16% of all adenosines in mammalian tissues and 0.015–0.054% in mammalian cell lines [94]. The m1A mostly occurs in the 5’UTR region with a GC-rich sequence near the translation initiation site (TIS) (Fig. 1b) [95]. The m1A enriched region suggests its potential function in stabilizing mRNA structure and translation [96]. In the CDS region of mRNA, m1A has been reported to disrupt Watson–Crick base pairing, thus impairing protein synthesis and altering mRNA structural stability [97]. In addition, m1A may change the secondary structure of mRNA 5’UTR region by introducing positive charges, resulting in increased accessibility of the translation machinery [98].

tRNA Methyltransferases (TRMTs) including TRMT10C, TRMT61B, TRMT6, and TRMT61A can add a methyl group at the N1 position of adenosine on targeted RNA (Figs. 1c, 2c) [99–101]. TRMT6/TRMT61A complex is mainly distributed in the cytosol, whereas TRMT10C/TRMT61B complex are mitochondrial methyltransferases since m1A also presents in 5′UTR of mitochondrial transcripts beside tRNA, rRNA, mRNA [102, 103]. Compared with mRNA, m1A is highly abundant in tRNAs [104]. The methylation of m1A at site 58 (m1A58) in tRNA can be catalyzed by the TRMT6/TRMT61A methyltransferase complex [105]. The m1G9 of mitochondrial (mt) tRNAs can be modified by TRMT10C, whereas m1A58 of mt tRNA-Leu (UUR) is modified by TRMT61B, respectively [106]. Meanwhile, the m1A947 of mt-16S rRNA is written by TRMT61B [107, 108].

m1A has been demonstrated to be erased by demethylases including ALKBH1 and ALKBH3 (Figs. 1c, 2c) [109]. ALKBH3 is a dealkylase, which is also considered as an alkylation damage repair enzyme [110]. It demethylates m1A and 3-methylcytosine (m3C) in RNA and single-stranded DNA [111]. ALKBH1 is responsible for demethylating m1A58 in tRNA [112]. ALKBH7 is an eraser that demethylates m1A in Ile and Leu1 pre-tRNA in the mitochondria (Figs. 1c, 2c) [113]. FTO, the m6A eraser mentioned above, can also remove m1A methylation (Fig. 2c) [114].

It has been indicated that YTH domain family (YTHDF1, YTHDF2, YTHDF3) can interact with m1A-carrying RNA, and are thereby jargonized as “readers” (Fig. 1c) [115]. Among these enzymes, YTHDF1 enables m1A-containing RNA to enter highly efficient translation, whereas YTHDF2/3 regulates the decay and stability of targeted RNA (Fig. 2c) [116].

### N7-methylguanosine (m7G)

The N7-methylguanosine (m7G) modification refers to adding a methyl group at the 7^th^ position of the guanosine nucleotide in RNA molecules. The m7G modification commonly locates at the 5’ caps of eukaryotic mRNA or internally within mRNA, tRNA, rRNA and miRNA, among which tRNA is the most abundant substrate of m7G modification (Fig. 1a-b) [117, 118]. In humans, the m7G modification on tRNA variable loop is catalyzed by the Methyltransferase-like 1 (METTL1) / WD repeat domain 4 (WDR4) complex (Figs. 1c, 2d) [119–121]. METTL1 binds with WDR4 to modulate mRNA translation through its effect on tRNA and ribosome biogenesis (Fig. 2d) [122]. METTL1 functions as a m7G catalytic component while WDR4 acts as a METTL1 corresponding cofactor that plays a stabilizing role (Fig. 2d) [123]. Through cellular, biochemical and structural studies of human METTL1-WDR4, Li et al. recently showed that WDR4 served as a scaffold for METTL1 and the tRNA T-arm [124]. They also revealed that the predicted disordered METTL1 N-terminus was part of the catalytic pocket, where the METTL1 N-terminal S27 phosphorylation inhibited methyltransferase activity by disrupting the catalytic center [124]. Moreover, mutations in METTL1/WDR4 complex are associated with developmental disorders such as primordial dwarfism and brain malformation [125].

WBSCR22 and tRNA methyltransferase activator subunit 11–2 (TRMT112) were identified as a methyltransferase complex for 18S rRNA m7G, which was involved in the processing and maturation of pre‐rRNA as well as 40S ribosome subunit biogenesis (Figs. 1c, 2d) [126, 127]. WBSCR22 is localized on chromosome 7 (7q11.23) and contains a nuclear localization signal and a common SAM binding motif [128]. TRMT112 acts as a cofactor for WBSCR22, because accessory proteins are required to enhance the stability and activity of several methyltransferases (Fig. 2d) [129]. It has been reported that TRMT112 is the accessory partner of WBSCR22 [129]. The m7G modification at the 5’ cap of mRNA is catalyzed by RNA guanine-7-methyltransferase (RNMT) and RNMT-Activating Mini protein (RAM) complex, which further stabilizes the nascent mRNA and protects from exonuclease attack (Figs. 1c, 2d) [130]. Existing articles confirm that the RNA nuclear export and efficient cap-dependent mRNA translation both rely on RNMT and its cofactor RAM (Fig. 2d) [131, 132].

However, specific “erasers” or “readers” that remove or recognize m7G modification have not yet been reported [25]. Current studies have demonstrated that m7G cooperates with the internal m6Am modification to regulate global RNA alternative splicing in human diseases [133]. Meanwhile, the m7G cap adjacent to the m6Am modification protects RNA stability from decay (Fig. 2d) [134]. If there already exists a 2′-O-dimethyl-adenosine (Am) after the m7G modification, the phosphorylated CTD interacting factor 1 (PCIF1), an m6Am methyltransferase, often catalyzes the Am site to form an m6Am modification (Fig. 2d) [134, 135]. The stability of most m6Am-marked RNA transcripts is unchanged in PCIF1 KO cells, it is thus unclear whether m6Am potentially regulates mRNA stability under particular conditions such as stress or differentiation [136]. Many studies are needed regarding m6Am mechanism and functional consequences. Meanwhile, METTL4 can be another m6Am methyltransferase that adds m6Am modification at internal sites of U2 small nuclear RNA (snRNA) containing an m7G-modified cap (Fig. 2d) [133]. To maintain a dynamic and reversible m6Am modification process, FTO undertakes the responsibility of removing such m6Am modifications (Fig. 2d) [137]. FTO is known to demethylate multiple types of RNA modifications, including m6A, m1A and m5C; whether it also functions as a m7G eraser remains to be determined [138].

### Pseudouridine (Ψ)

Pseudouridine (Ψ), a C–C glycosyl isomer, is produced by the isomerization of specific uridine (U) bases [139]. The pseudouridylation process is catalyzed by Ψ synthases [140]. Ψ can be observed in all stable RNAs including tRNAs, rRNAs, snRNAs and recently also in mRNAs (Fig. 1a-b) [141]. The base-specific pseudouridylation is mediated by small nucleolar RNA (snoRNA)-dependent or RNA-independent mechanism [140], relying on distinct Ψ synthases. The snoRNA-dependent pathway depends on a small nucleolar ribonucleoprotein (snoRNP) complex whereas RNA-independent mechanism requires pseudouridine synthase (PUS) family enzymes (Figs. 1c, 2e) [142]. Pseudouridine synthase, in combination with three core proteins (NOP10, GAR1 and NHP2), forms a ribonucleoprotein (RNP) complex (Fig. 2e) [143]. The RNP complex must be guided by ncRNAs known as box H/ACA snoRNAs to the appropriate modification sites, together consisting of the snoRNP complex (Fig. 2e) [144]. The catalytic portion of the snoRNP complex is the pseudouridine synthase dyskerin, a nucleolar protein encoded by the DKC1 gene at Xq28 (Fig. 1c, 2e) [145]. Furthermore, dyskerin (Cbf5 in yeast) is related to telomere activity and mRNA splicing (Fig. 2e) [146]. In eukaryotes, the PUS enzymes, including Pus1, Pus2, Pus4 and Pus7, are involved in mRNA pseudouridylation and independently modify uridine by recognizing specific sequences and/or secondary structural elements of the targeted RNA (Fig. 2e) [140, 147]. In general, the Ψ base modification stabilizes RNA, improves base-stacking and modulates transcription.

### Adenosine-to-inosine editing (A-to-I editing)

A-to-I is a site-specific alteration catalyzed by adenosine deaminases acting on RNA (ADAR) enzymes (Fig. 1) [148]. ADAR contains a C-terminal catalytic domain (deaminase domain) and an N-terminal RNA-binding domain that binds double-stranded RNA (dsRNA) between an editing-site-containing sequence and an up/downstream editing complementary sequence (Fig. 2f) [149]. The conversion of adenosines into inosines is then accomplished by hydrolytic deamination [150]. Because inosine is often “read” as guanosine by translation machinery, the conversion of nucleotides potentially transforms RNA splicing, maturation, miRNA targeting and the ultimate translated amino acid sequence [151]. There exist three mammal ADAR enzymes: ADAR1 and ADAR2 are catalytically active and extensively expressed, whereas ADAR3 is enzymatically inactive and displays brain-specific expression [152, 153]. ADAR1 comprises two isoforms: a 150-kDa isoform (p150) that is interferon-inducible and a 110-kDa isoform (p110) that differs at the N-terminus (Fig. 5e) [154]. The p110 form is initiated from a downstream methionine as a result of skipping the upstream exon-containing methionine, whereas the p150 isoform is generated from an IFN-inducible promoter [148]. Actually, A-to-I RNA editing is equivalent to A-to-G cDNA conversion, thus inducing altered RNA splicing sites, perturbed dsRNA structures and amino acid substitutions (Fig. 2f) [155].

### N4-acetylcytidine (ac4C)

Ac4C, a conserved chemical modification, is currently the only known RNA acetylation event that occurs on rRNA, tRNA and mRNA in eukaryotic RNA (Fig. 1) [15]. Almost five decades ago, ac4C modification was first identified in yeast tRNA [156]. N-acetyltransferase 10 (NAT10) has been identified as the main ac4C “writer”, an enzyme with both acetyltransferase activity and RNA binding ability (Fig. 1c) [157]. NAT10 was originally found to regulate telomerase activity and rRNA transcription in the nucleolus, thus playing a role in delaying aging, preventing osteoporosis and promoting tumor metastasis [158]. In 2014, the *Saccharomyces cerevisiae* homolog of human NAT10, was uncovered to catalyze the ac4C-1773 of 18S rRNA, promoting the formation of pre-18S rRNA [159]. Subsequently, human NAT10 was reported to catalyze the ac4C-1842 of 18S rRNA [160]. NAT10-mediated ac4C modification affects multiple biological processes, including mRNA stability and translation efficiency (Fig. 2g) [161]. Although the identity of the ac4C “readers” and “erasers” are still undetermined, a few studies have focused on a candidate “eraser” Sirtuin 7 (SIRT7) (Figs. 1c, 2g) [162]. SIRT7 was considered as an RNA deacetylate in vitro because of the observation of elevated endogenous ac4C levels on snoRNA in a SIRT7 deficient cell line [163]. Therefore, SIRT7 being a promising “eraser” of ac4C still needs further verification with an increasing number of studies [163]. More functions of ac4C in various biological processes as well as detailed mechanisms regarding ac4C addition, removal and recognition is an interesting field for future studies in RNA modification.

## Dysregulation of RNA modifications in disease pathologies

**Fig. 3:**
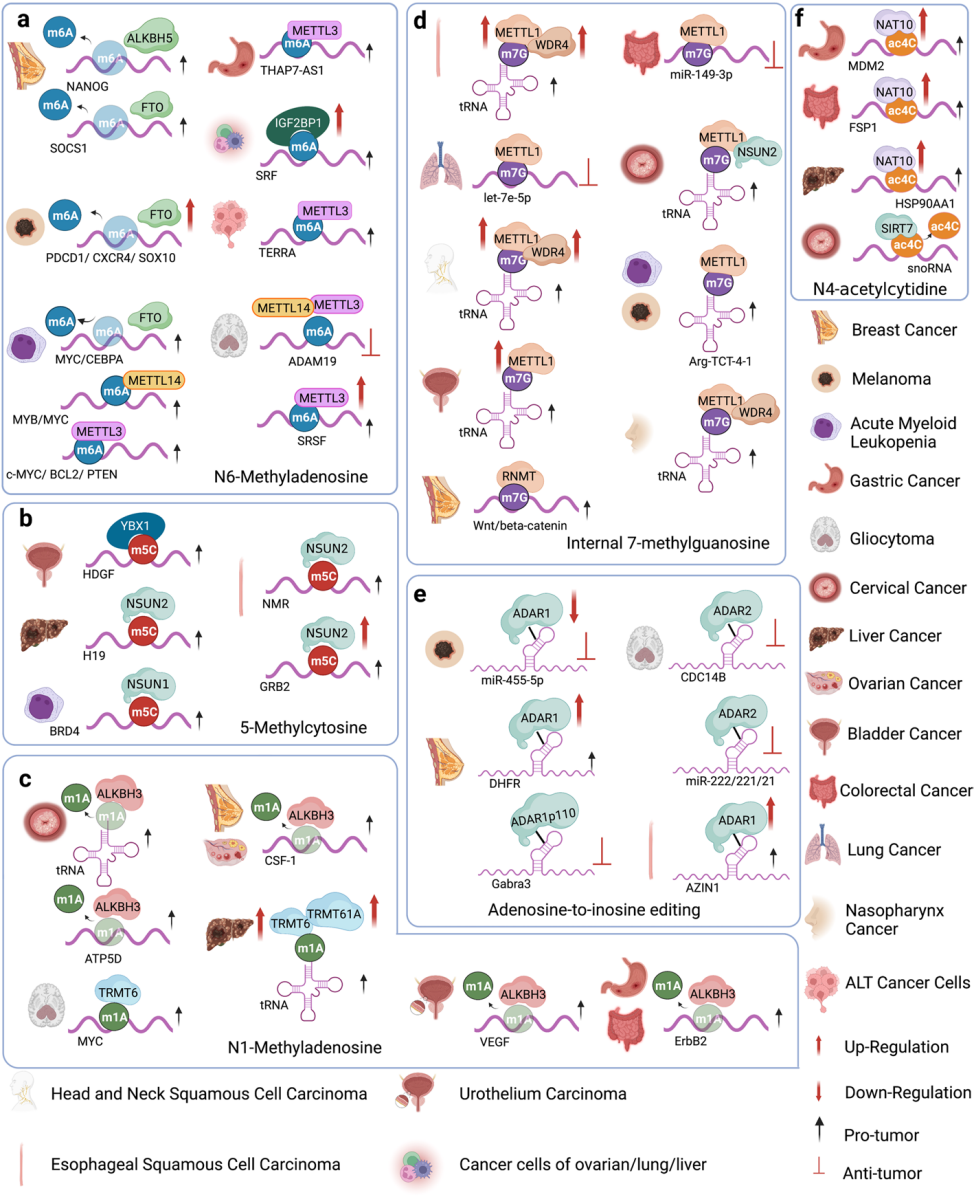
The regulation of different RNA modifying enzymes in various tumors. **a** The m6A-associated RNA modifying enzymes involved in multiple tumors and their respective substrate RNAs. **b** The m5C-associated RNA modifying enzymes involved in multiple tumors and their respective substrate RNAs. **c **The roles of m1A-associated RNA modifying enzymes in tumors modulating various substrate RNAs. **d** The roles of m7G-associated methyltransferases regulating tRNAs and mRNAs in multiple tumors. **e** The roles of A-to-I editing modifiers in regulating substrate double-stranded RNAs in multiple tumors. **f** Regulation of substrate RNAs by ac4C modifiers, NAT10 and SIRT7, in various tumors

Dysregulation in RNA modifying enzymes has been reported in various disease models, including multiple cancer types (Fig. 3, Table 1), neurological disorders, cardiovascular diseases, metabolic diseases, as well as genetic and developmental disorders.

**Table 1:** Roles of RNA modifiers in cancer, their substrates and targeted drugs

Modification type	Cancers	Regulators	Role in cancers	Function in cancers	Substrate / *targeted drug*	PMID
m6A	Gastric Cancer	METTL3	Tumor Promotion	Enhance the expression of THAP7-AS1	THAP7-AS1	34608273
m6A	Glioblastoma	METTL3	Tumor Promotion	Maintain SRSFs’ protein expression	SRSFs	31530567
m6A	Glioblastoma	METTL3/METTL14	Tumor Inhibition	Depress GSC growth, self-renewal	ADAM19	28297667
m6A	Acute Myelogenous Leukemia	METTL3	Tumor Promotion	Reduce cell differentiation and apoptosis	c-MYC, BCL2, PTEN	28920958
m6A	AML Cancer Cell	METTL3	Tumor Promotion	Stabilize TERRA	TERRA	36399511
m6A	Lung Cancer	METTL3	Tumor Promotion	Promote translation of BRD4	BRD4	30232453
m6A	Acute Myelogenous Leukemia	METTL14	Tumor Promotion	Modify MYB/MYC mRNA	MYB/MYC	29290617
m6A	Melanoma	FTO	Tumor Promotion	Anti-PD-1 resistance	PDCD1, CXCR4, SOX10	31239444
m6A	Leukemia	FTO	Tumor Promotion	Facilitate mRNA stability of MYC/CEBPA	MYC, CEBPA / *R-2HG*	29249 359
m6A	Various Cancer Cell	FTO	Tumor Promotion	Inhibit the abundance of SOCS1	SOCS1 / *18097*	35256950
m6A	Breast Cancer	ALKBH5	Tumor Promotion	Increase NANOG mRNA/protein and the BCSC phenotype	NANOG	27001847
m6A	Various Cancer Cell	IGF2BP1	Tumor Promotion	Prevent the decay of the SRF mRNA	SRF	30371874
m6A	Melanoma	YTHDF1	Tumor Promotion	Impair presentation of tumor antigens	YTHDF1	30728504
m5C	Hepatocellular Carcinoma	NSUN2	Tumor Promotion	Mediate m5C modification of H19	H19	32978516
m5C	Triple Negative Breast Cancer	NSUN2/NSUN6	Tumor Promotion	Regulate RNA degradation/cell adhesion	–-	33928086
m5C	ESCC	NSUN2	Tumor Promotion	Methylate NMR	NMR	29763634
m5C	ESCC	NSUN2	Tumor Promotion	Stabilize GRB2	GRB2	34345012
m5C	Gastric Cancer	NSUN2	Tumor Promotion	Promote the cell proliferation, migration	Stabilized by SUMO2/3	34504059
m5C	Acute Myelogenous Leukemia	NSUN1	Tumor Promotion	Motivate 5-AZA-resistant of BRD4	BRD4 / *5-AZA*	29563491
m5C	Lung Cancer	NSUN3/NSUN4	Tumor Promotion	Affect infiltration of immune cells	–-	34195072
m5C	Pancreatic Cancer	NSUN6	Tumor Inhibition	Regulate cell proliferation, tumor growth	–-	33418496
m5C	Bladder Cancer	YBX1	Tumor Promotion	Stabilize HDGF	HDGF	31358969
m1A	NSCLC	ALKBH3	Tumor Promotion	Reduce cell cycle arrest or apoptosis	–-	28479246
m1A	Prostatic Cancer	ALKBH3	Tumor Promotion	As prostate cancer antigen-1 with PCA-1	–- / *HUHS015*	24461353
m1A	Cervical Cancer Cell	ALKBH3	Tumor Promotion	Promote the cell proliferation, migration	tRNA	30541109
m1A	Breast / Ovarian Cancer	ALKBH3	Tumor Promotion	Regulate the CSF-1 mRNA stability	CSF-1	30342176
m1A	Urothelium Carcinoma	ALKBH3	Tumor Promotion	Induce VEGF expression	VEGF	22850567
m1A	Gastrointestinal Tumor	ALKBH3	Tumor Promotion	Facilitate ErbB2 and AKT1S1 expression	ErbB2	31352195
m1A	Cervical Cancer Cell	ALKBH3	Tumor Promotion	Facilitate the translation elongation of ATP5D	ATP5D	35867754
m1A	Abdominal Aneurysm	YTHDF3	Tumor Promotion	Promote aortic inflammation	–-	35620523
m1A	Trophoblast	YTHDF3	Tumor Inhibition	Promote IGF1R mRNA degradation	IGF1R	32194978
m1A	Glioblastoma	TRMT6	Tumor Promotion	Promote the cell proliferation, migration	MYC	34631793
m1A	Hepatocellular Carcinoma	TRMT6/TRMT61A	Tumor Promotion	Increase PPARδ translation	tRNA	34728628
m7G	Hepatocellular Carcinoma	METTL1	Tumor Promotion	Promote the cell proliferation, migration	–-	31463732
m7G	ESCC	METTL1/WDR4	Tumor Promotion	Motivate RPTOR/ULK1/ autophagy	tRNA	35304469
m7G	NSCLC	METTL1	Tumor Inhibition	Augment let-7 miRNA processing	let-7e-5p	31031083
m7G	Colorectal Cancer	METTL1	Tumor Inhibition	Enhance the cytotoxic effects of cisplatin	miR-149-3p / *cisplatin*	31866582
m7G	HNSCC	METTL1/WDR4	Tumor Promotion	Induce PI3K/AKT/mTOR signaling	tRNA	35179319
m7G	Bladder Cancer	METTL1	Tumor Promotion	Regulate the translation of EGFR/EFEMP1	tRNA	34936728
m7G	Cervical Cancer Cell	METTL1/NSUN2	Tumor Promotion	Modulate 5-FU sensitivity in HeLa	tRNA / *5-FU*	25233213
m7G	Various Cancer Cell	METTL1	Tumor Promotion	Drive oncogenic transformation	tRNA	34352207
m7G	Nasopharynx Cancer	METTL1/WDR4	Tumor Promotion	Improve translation efficiencies of mRNAs	tRNA	35217794
m7G	Glioblastoma	WBSCR22	Tumor Promotion	Induce Akt /GSK3β phosphorylation	–-	32380188
m7G	Colorectal Cancer	WBSCR22	Tumor Promotion	Induce cellular resistance to oxaliplatin	–- / *oxaliplatin*	29133897
m7G	Pancreatic Cancer	WBSCR22/TRMT112	Tumor Inhibition	Negatively regulate ISG15	–-	35088887
m7G	Breast Cancer Cell	RNMT	Tumor Promotion	Elevate mRNA cap methylation of Wnt	Wnt/beta-catenin	27899423
A To I	Melanoma	ADAR1	Tumor Inhibition	Suppress of melanoma growth, metastasis	miR-455-5p	25686251
A To I	Breast Cancer	ADAR1	Tumor Promotion	Up-regulate DHFR expression	DHFR	28188287
A To I	ESCC	ADAR1	Tumor Promotion	Drive the aggressive tumor behavior	AZIN1	24302582
A To I	Breast Cancer	ADAR1-p110	Tumor Inhibition	Edit Gabra3’ and reverse its function as a tumor suppressor	Gabra3	26869349
A To I	Astrocytoma	ADAR1/2/3	Tumor Inhibition/Promotion	ADAR1 forms heterodimer with ADAR2 and interferes with ADAR2 editing activity	–-	18178553
A To I	Glioblastoma	ADAR2	Tumor Inhibition	Increase CDC14B expression	CDC14B	22525274
A To I	Glioblastoma	ADAR2	Tumor Inhibition	Reduce miR-222/221/21 expression	miR-222/221/21	25582055
A To I	Astrocytoma	ADAR2	Tumor Inhibition	Inhibit tumor growth	–-	23697632
Ψ	Glioblastoma	DKC1(Dyskerin)	Tumor Promotion	Stimulate glioma cell growth	–-	30847721
Ψ	Hepatocellular Carcinoma	DKC1(Dyskerin)	Tumor Promotion	Facilitate MYC and MKI67 expression	MYC/MKI67	22912812
Ψ	Hypophysoma	DKC1(Dyskerin)	Tumor Inhibition	Maintain the translation of p27	–-	20587522
Ψ	Prostatic Cancer	DKC1(Dyskerin)	Tumor Promotion	Predictor of prostate cancer	rRNA	31511832
Ψ	Melanoma and Breast Cancer	mPus1p	Tumor Promotion	Enhance mRARgama-mediated transcription	–-	15327771
Ψ	Various Cancer Cell	PUS10	Tumor Inhibition	Induce the TRAIL sensitivity of tumor cells	–-	28981101
ac4C	Pancreatic Cancer	NAT10	Tumor Promotion	Maintain the stability of downstream cancer mRNA	–-	35978332
ac4C	Gastric Cancer	NAT10	Tumor Promotion	Stabilize MDM2 mRNA	MDM2	36609449
ac4C	Hepatocellular Carcinoma	NAT10	Tumor Promotion	Maintain the stability of HSP90AA1	HSP90AA1	36765042
ac4C	Colorectal Cancer	NAT10	Tumor Promotion	Affect FSP1 mRNA stability and ferroptosis	FSP1	36209353
ac4C	Breast Cancer Cell	SIRT7	–-	Deacetylate ac4C on snoRNA	snoRNA	–-

### Cancer

Cancer is a major public health problem that affects people’s life all over the world. It is a complex disease with many subtypes and variations and can be further classified based on specific characteristics, such as the stage and grade. Treatment approaches and outcomes vary depending on the type and stage of cancer. Many studies have provided evidence suggesting that dysregulation of RNA modifications or RNA modifying enzymes contributes to human cancers. The list of important RNA modifying enzymes that play essential roles in cancer keeps expanding as research progresses in this field (Table 1).

#### m6A dysregulation in cancer

m6A dysregulation has been implicated in various aspects of cancer development. Dysregulation of m6A may lead to altered RNA stability, disrupted splicing patterns, disrupted RNA processing and maturation, as well as altered efficiency and accuracy of translation, resulting in aberrant expression of oncogenic or the tumor-suppressive genes, thus contributing to cancer initiation and progression [164]. m6A dysregulation may also contribute to epigenetic alterations in cancer cells. Abnormal m6A modification patterns can affect the accessibility of chromatin and DNA methylation, consequently influencing gene expression and cellular phenotype in cancer cells. Furthermore, dysregulation of m6A may affect cancer stem cell (CSC) maintenance, differentiation, and tumorigenic potential, thereby impacting tumor growth and therapy resistance [54].

##### METTL3-METTL14

Depending on the different m6A-modified RNA targets, the role of the METTL3/14 complex may be contradictory in some cancer models, as opposite effects have been reported in different studies [165].

Most research studies on the m6A writer METTL3 or METTL14 indicate their oncogenic roles in cancer. For example, METTL3 is abundantly expressed in Acute myelogenous leukemia (AML) and has been identified as a crucial gene for AML cell proliferation by a whole-genome CRISPR dropout screening approach [27, 166]. METTL3 depletion in human hematopoietic stem/progenitor cells (HSPCs) increased cell differentiation and reduced cell proliferation [166]. Leukemic cells without METTL3 also failed to establish leukemia mouse xenograft [27]. At the molecular level, m6A modification mediated by METTL3 promoted the translation of different oncogenic targets including PTEN, c-MYC and BCL2 in the human AML MOLM-13 cell line, whereas METTL3 loss led to an increase in AKT phosphorylation, contributing to the differentiation phenotype (Fig. 3a) [166]. METTL3 can also bind to the transcriptional start site region of active genes in presence of CEBPZ in a METTL14-independent manner, where it induces m6A modification and enhances translation of genes that are necessary for AML [27]. The METTL3 partner protein METTL14 was highly expressed in normal HSPCs and various AMLs, where it exerted its oncogenic role by regulating MYB and MYC mRNA through m6A modification (Fig. 3a) [73].

METTL3 expression was also elevated in glioblastoma stem-like cells (GSCs) and was attenuated during differentiation [167]. This elevation of METTL3 was associated with clinical aggressiveness of malignant gliomas [168]. Glioblastoma (GBM) is the most prevalent and malignant primary brain tumor as patients often recur after chemotherapy treatment due to an undifferentiated cancer stem cell (CSC) population that is therapeutic resistant [42]. METTL3 enhanced the stability of SOX2 mRNA through m6A modification and was crucial for GSC resistance to γ-irradiation and DNA repair [167]. Downregulating METTL3 suppressed GSC proliferation and self-renewal by decreasing m6A modification of serine- and arginine-rich splicing factors (SRSFs), leading to YTHDC1-dependent SRSF mRNA decay and decreased translation (Fig. 3a) [168].

Besides AML and glioblastoma, oncogenic roles were also reported for METTL3 in multiple other cancer types. Elevated METTL3 expression was observed in human lung adenocarcinoma, where METTL3 played an essential role in promoting cancer cell survival, proliferation and invasion [47]. Cytoplasmic METTL3 directly promoted translation of oncogenes, including the Hippo pathway effector TAZ and the epidermal growth factor receptor (EGFR), by interacting with the translation initiation machinery [47]. Choe et al.further uncovered that METTL3 interacted with the eukaryotic translation initiation factor 3 subunit h (eIF3h), together enhancing translation of oncogenic mRNAs, including Bromodomain-containing protein 4 (BRD4), in human primary lung tumors and promoting tumorigenicity [169]. In hepatocellular carcinoma (HCC) patients, METTL3 overexpression correlated with poor prognosis [170]. METTL3 is responsible for the m6A-mediated suppressor of cytokine signaling 2 (SOCS2) mRNA degradation [170]. METTL3 promotes liver cancer cell epithelial-mesenchymal transition (EMT) by triggering polysome-mediated translation of Snail mRNA through m6A modification of Snail CDS region [171]. In gastric cancer, METTL3-mediated m6A modification of THAP7-AS1 enhanced its expression, which promoted cell progression by improving CUL4B entry into the nucleus to repress miR-320a and miR-22-3p transcription (Fig. 3a) [172]. METTL3-mediated m6A modification on the sub-telomeric regions of telomeric repeat-containing RNA (TERRA) led to R-loop formation and promoted homologous recombination (HR), which was essential for the alternative lengthening of telomeres (ALT) pathway and telomere stability in cancer cells (Fig. 3a) [173].

Tumor suppressor functions of METTL3-METTL14 complex have also been reported the in several cancer models. For example, Cui et al.showed that the METTL3-METTL14 complex inhibited GSC self-renewal and tumorigenesis by regulating mRNA m6A enrichment and expression of genes with critical oncogenic functions, such as a disintegrin and metalloproteinase domain-containing protein 19 (ADAM19) (Fig. 3a) [174]. METTL14 was shown to suppress liver cancer metastasis by interacting with the microprocessor protein DGCR8 and promoting microRNA 126 maturation in a m6A-dependent manner [175]. In endometrial cancer, m6A methylation reduction by either METTL14 mutations or METTL3 downregulation led to the stabilization of mRNAs encoding members of the AKT pathway, decreased PHLPP2 expression and increased mTORC2 expression, leading to AKT pathway activation and cell proliferation [176].

##### METTL16

The role of the U6 spliceosomal snRNA methyltransferase METTL16 in cancer has not yet been well studied. METTL16 is crucial for AML cell proliferation and regulates MAT2A mRNA splicing to maintain SAM homeostasis [6, 177]. METTL16 is also important for the maturation of the metastasis-associated lung adenocarcinoma transcript 1 (MALAT1) lncRNA [178], which may act as either an oncogene or a tumor suppressor depending on the cancer type [179]. The anti-proliferative role of METTL16 in *Caenorhabditis elegans* indicates that the METTL16-MALAT1 complex may be crucial for the oncogenic activity of MALAT1 [178]. Further studies are still necessary to unravel the specific roles of METTL16 in cancer.

##### FTO

The role of the m6A eraser FTO in cancer was first demonstrated in melanoma, where specific FTO variants were associated with increased melanoma risk in a BMI-independent manner [38]. A more recent study showed that FTO promoted melanoma tumorigenesis as well as tumor resistance to interferon gamma (IFNγ) and anti-PD-1 treatment by demethylating m6A from crucial pro-tumorigenic mRNAs, including programmed cell death 1 (PDCD1), SRY-Box Transcription Factor 10 (SOX10) and CXC-chemokine receptor 4 (CXCR4), leading to increased mRNA stability (Fig. 3a) [180]. FTO also plays a crucial oncogenic role in AML, where it regulates expression of targets such and as ankyrin repeat retinoic acid receptor-α (RARA) and SOCS box-containing 2 (ASB2) by reducing their mRNA transcript m6A levels, thus enhancing cell transformation and leukemogenesis [181]. This mechanism seemed to be specific for isocitrate dehydrogenase 1 (IDH1) wild-type leukemia cells [6, 182]. Leukemia-associated IDH1/2 mutations induce a neomorphic enzymatic function that converts α-ketoglutarate to 2-hydroxyglutarate [183]. This conversion is generally pro-tumorigenic, except that in AML and glioma cells this oncometabolite inhibits FTO enzymatic activity and m6A accumulation on FTO targets, thus eliciting tumor suppressing effects *in vitro* and *in vivo* [57]. In clear cell renal cell carcinoma (ccRCC), FTO seemed to have demonstrated contradictory effects. Some data demonstrated an association of decreased FTO expression with aggressive clinical features and shorter overall survival in ccRCC patients. In contrast, others revealed that FTO inhibition reduced the survival and proliferation of VHL-deficient ccRCC cells both in vitro and in vivo [184].

##### ALKBH5

Primary glioblastoma samples and GSCs expressed higher levels of the m6A demethylase ALKBH5, which correlated with worse patient prognosis [185]. ALKBH5 demethylated the nascent transcripts of forkhead box protein M1 (FOXM1), enhancing its expression [185]. Downregulating ALKBH5 or disrupting the interaction between AKLBH5 and FOXM1 transcript by depleting the lncRNA antisense to FOXM1 (FOXM1-AS) both disrupted GSC tumorigenesis [185]. In breast cancer, hypoxia induced ALKBH5-mediated m6A demethylation of NANOG mRNA, leading to a stem cell phenotype (Fig. 3a) [186]. In melanoma and colon syngeneic mouse models, ALKBH5 attenuates tumor response to anti-PD-1 therapy by modulating Mct4/Slc16a3 expression, lactate content, as well as the composition of myeloid-derived suppressor cells and tumor-infiltrating Treg cells in the tumor microenvironment [46].

##### YTH-domain containing proteins

YTH-domain containing proteins are a group of proteins with a conserved RNA-binding domain known as the YTH (YT521-B homology) domain [187]. This domain enables these proteins to recognize and bind to specific RNA molecules [187]. The m6A reader YTHDF1 can play a pro-oncogenic role through its function in immune cells. Ythdf1-knockout mice showed an elevated anti-tumor response against melanoma xenografts due to increased antigen cross-presentation of YTHDF1-depleted dendritic cells [33]. Through recognition of m6A-marked transcripts, YTHDF1 promotes the translation of lysosomal cathepsins, inhibition of which enhance cross-presentation of dendritic cells antigen [33]. YTHDC2 is overexpressed in human colorectal cancers and contributes to colon tumor metastasis by unwinding the 5'-untranslated region (5'UTR) of mRNA, thus promoting HIF-1α translation [188]. The mRNA m6A reader YTHDF2 was overexpressed in AML and is required for AML tumorigenesis and progression [54]. YTHDF2 decreased the half-life of m6A-methylated transcript of diverse tumor necrosis factor receptor Tnfrsf2, which played a crucial role in apoptosis of leukemic stem cells (LSCs) [54]. YTHDF2 has also been reported to act as a tumor suppressor in colorectal cancer (CRC), melanoma and osteosarcoma. In gastric cancer (GC), liver cancer and lung cancer, YTHDF2 was found to be both upregulated and downregulated, suggesting that YTHDF2 may play a dual role as both an oncogene and tumor suppressor [184].

##### IGF2BPs

Insulin-like growth factor 2 messenger RNA binding proteins (IGF2BPs) specifically recognize m6A-modified RNAs through their KH domains [6]. These proteins are highly expressed upon malignant transformation in a broad range of cancer types and often correlate with poor patient prognosis [31], although their function may not always depend on m6A recognition. Nonetheless, IGF2BP1 has been shown in multiple cancer cell lines to stabilize the c-myc mRNA by interacting with the coding region instability determinant (CRD) in an m6A-dependent manner [189]. Furthermore, IGF2BP1 promoted serum response factor (SRF) expression in an m6A-dependent manner, enhancing transcription of SRF-target genes, including PDLIM7 and FOXK1, thereby promoting tumor cell growth and invasion (Fig. 3a) [53]. SRF/IGF2BP1-dependent genes also correlated with poor prognosis in ovarian, liver and lung cancer [53]. In summary, all m6A RNA modifiers have been implicated in cancer, where they are generally pro-tumorigenic with occasional tumor repressing roles depending on downstream target RNAs and cancer types.

#### m5C dysregulation in cancer

m5C is another RNA modification that has been implicated in cancer. Similar to m6A, dysregulation of m5C modification can lead to altered RNA stability, disrupted splicing patterns, altered epigenetic patterns [190]. Dysregulation of m5C can also disrupt proper RNA folding and alter RNA interactions with other molecules, impact the subcellular localization of specific RNAs, as well as affect the expression of immune-related genes and impact immune checkpoint regulation, potentially influencing the tumor microenvironment and immune evasion mechanisms [190].

Like m6A, most m5C modifiers are known for their oncogenic roles. NSUN1 was first identified as a proliferation nuclear antigen [191]. NSUN1 was later found to be overexpressed in prostate and lung cancer, where it correlated strongly with poor patient prognosis [192, 193]. NSUN2 was highly overexpressed in multiple tumor types either through amplification [194, 195] or DNA hypomethylation [9]. NSUN2 knockdown inhibited cell proliferation in NSUN2-overexpressing breast cancer and in MYC-driven squamous cell carcinoma [9, 194, 196]. It was reported that NSUN2 could cooperate with Y-box-binding protein 1 (YBX1), an m5C ‘reader’, to drive pathogenesis of human urothelial carcinoma of the bladder (UCB) by stabilizing oncogenic mRNAs, such as heparin-binding growth factor (HDGF), via m5C methylation (Fig. 3b) [91]. Notably, YBX1 has previously been reported to play oncogenic roles in multiple tumor types, including bladder cancer [65] and breast cancer [197]. In HCC, NSUN2 mediates the m5C modification of a tumor-related lncRNA H19, increasing its stability [198]. The high m5C methylation level and the H19 expression level in HCC tissues were closely associated with poor differentiation of HCC (Fig. 3b) [198]. For example, NSUN2 promotes tumor metastasis and cisplatin resistance by methylating NMR (also known as LINC01672) ncRNA, which in turn recruits BPTF and promotes the expression of matrix metalloproteinase 3 (MMP3) and MMP10 in esophageal squamous cell carcinoma (Fig. 3b) [199]. NSUN2 overexpression was linked to poor prognosis of esophageal squamous cell carcinoma (ESCC) patients, whereas its silencing suppressed in vivo tumorigenesis and progression of ESCC in Nsun2-KO mice [62]. Mechanistically, NSUN2 stabilized the mRNA of growth factor receptor-bound protein 2 (GRB2) by increasing its m5C modification, which was coregulated by a new m5C mediator, the lin-28 homolog B (LIN28B) protein (Fig. 3b) [62]. Bioinformatic analysis of m5C regulators (TRDMT1, NSUN1-7, DNMT1-2, DNMT3a/B, ALYREF and TBX1) in lung squamous cell carcinoma (LUSC) revealed that most of the m5C regulators were upregulated in LUSC compared with normal samples and were associated with poor prognosis [200]. Similar analysis by Huang and colleagues on 11 m5C RNA methylation regulators (NSUN2-7, DNMT1, DNMT3A/B, ALUREF and TET2) in breast cancer databases demonstrated that NSUN2 overexpression closely correlated to cell cycle signaling pathways, RNA polymerase, spliceosome, and RNA degradation, whereas NSUN6 depletion correlated to metabolism, extracellular matrix receptor interaction, and cell adhesion [184]. NSUN2 was also upregulated in GC, where it promoted GC cell proliferation, migration, and invasion possibly by mediating the m5C methylation of oncogenes such as PIK3R1 and PCYT1A [201].

By analyzing 382 tumors and 362 normal specimens from pancreatic cancer (PC) patients, Yang and colleagues characterized NSUN6 as an important factor regulating PC cell proliferation and suppressing PC development [202].

#### m1A dysregulation in cancer

m1A is a relatively less studied RNA modification that has only gained attention in recent years. Although its role in cancer is less extensively studied compared to m6A and m5C, emerging evidence suggests that m1A dysregulation may also contribute to cancer development and progression. Dysregulation of m1A in cancer may affect the stability of specific RNA molecules, impact the translation of specific mRNA transcripts, disrupt RNA processing and maturation, perturb RNA–protein interactions, as well as disturb the balance between m1A deposition and removal, leading to cancer development and progression [203]. Bioinformatic analysis of N1-methyladenosine (m1A) regulators (TRMT6/61A, RRP8, ALKBH1/3, YTHDF1-3, YTHDC1) in LUSC revealed that most of the m1A regulators were significantly upregulated in cancer tissues compared to normal samples [200].

##### TRMT6

Members of the m1A methyltransferase complex, TRMT6 and TRMT61A, were overexpressed in advanced HCC tissue and correlated negatively with HCC survival [204]. TRMT61A/TRMT6-mediated tRNA m1A methylation drove liver CSC self-renewal and tumorigenesis by elevating PPARδ translation and triggering cholesterol synthesis to activate Hedgehog signaling (Fig. 3c) [204]. TRMT6 also predicted poorer prognosis in glioma and promoted glioma cell proliferation, migration, and invasion by regulating cell cycle, MYC, TGF-β, PI3K-AKT, NOTCH, and MTORC1 pathways (Fig. 3c) [205].

##### ALKBH3

The m1A eraser ALKBH3 was originally identified as a prostate cancer antigen that showed high mRNA expression in prostate carcinoma [206]. Later research revealed that ALKBH3 plays crucial roles in cancer cell proliferation as well as metastasis. For example, ALKBH3 is important for the repair of DNA alkylation damage [87], indicating a possibility that ALKBH3 function in cancer may be independent of its catalytic activity as m1A demethylase. In human lung cancers, particularly in lung adenocarcinomas and squamous cell carcinomas, ALKBH3 was overexpressed and was significantly correlated to recurrence-free survival [207]. Silencing ALKBH3 led to cell cycle arrest and senescence in vitro and peritoneal tumor growth and dissemination in vivo, possibly by inducing the expression of p21^WAF1/Cip1^ and p27^Kip1^ in lung adenocarcinoma cells [207]. In human urothelial carcinoma cells, ALKBH3 contributed to cancer survival, invasion and angiogenesis by mediating the level of NADPH oxidase-2 (NOX-2)-generated reactive oxygen species (ROS), as well as the expression levels of tumor necrosis factor-like weak inducer of apoptosis (Tweak), Vascular endothelial growth factor (VEGF) and fibroblast growth factor-inducible 14 (Fn14) (Fig. 3c) [208]. ALKBH3-mediated m1A, m3C and m6A demethylation can promote protein synthesis and cell proliferation in PANC-1 human pancreatic cancer cells [209]. Overexpressed human ALKBH3 in NSCLC significantly correlated with poor prognosis [210]. ALKBH3 knockdown induced NSCLC cell cycle arrest or apoptosis in a TP53-dependent manner [210]. ALKBH3 promoted cancer cell proliferation, migration and invasion by demethylating tRNAs, generating tRNA-derived small RNAs (tDRs) that prevent Cytochrome C-triggered apoptosis in various cancer cell lines (Fig. 3c) [84]. Furthermore, ALKBH3 was also reported to promote ovarian and breast cancer invasiveness by demethylating m1A and stabilizing the colony-stimulating factor 1 (CSF1) mRNA without affecting cell proliferation or migration (Fig. 3c) [211]. By analyzing TCGA data of patients with five different types of gastrointestinal (GI) cancers from cBioPortal, Zhao and colleagues demonstrated ALKBH3 knockdown decreased the expression of both AKT1S1 and ErbB2 (Fig. 3c) [212]. Gene Ontology analysis also indicated that m1A downstream genes were linked to cell proliferation [212]. ALKBH3 could positively regulate the glycolysis of cancer cells by demethylating an important adenosine 5’-triphosphate synthase subunit, ATP5D, whose m1A modification negatively regulated its own translation elongation and mRNA release from ribosome complex (Fig. 3c) [213].

##### YTHDF3

Regulation of m1A modification significantly correlated with the pathogenesis of human Abdominal Aortic Aneurysm (AAA), where the m1A reader, YTHDF3, modulated macrophage polarization and regulated the expression of key AAA-related target genes, including signal transducer and activator of transcription 3 (STAT3), CD44, ITGB1 and mTOR [214].

#### m7G dysregulation in cancer

By manipulating the metabolism of various RNA species, including mRNA, rRNA, miRNA, and tRNA, m7G actively participates in biological and pathological processes of cancer cells [118]. Increasing evidence suggests an important role for m7G in human cancer, where dysregulated m7G levels are closely related to tumorigenesis and progression by regulating the expression of multiple oncogenic and tumor-suppressing genes [118].

##### METTL1

In various cancer types, METTL1 inactivation through phosphorylation at Ser27 by protein kinase B (PKB) α and ribosomal S6 kinase (RSK) was responsible for driving tumor invasion and metastasis [215]. In human colon and lung cancer cells, METTL1 was required for m7G modification of the tumor suppressor microRNA let-7e to maintain high levels of mature let-7e, whose downregulation leads to high mobility group AT-hook 2 (HMGA2) overexpression (Fig. 3d) [216]. METTL1 loss in these cancer cells resulted in elevated migration potential *in vitro* [216]. METTL1 was downregulated in cisplatin-resistant colon cancer cells compared to their paired cisplatin-sensitive colon cancer cells [217]. Overexpressing METTL1 enhanced chemosensitivity of cells to cisplatin treatment by regulating miR-149-3p/S100A4/p53 axis (Fig. 3d) [217].

METTL1 may also promote tumor progression in some other conditions. For example, it has been considered as a potential driver in human glioblastoma due to its amplification and correlation with poor prognosis [218]. It was also necessary for AML cell viability [27]. Moreover, METTL1 overexpression in HCC correlated with low expression of the tumor suppressor PTEN, increased tumor size, tumor vascular invasion, higher serum Alpha-Fetoprotein (AFP) level, and poor prognosis [219]. METTL1-mediated m7G modification on Arg-TCT-4–1 tRNA induced oncogenic cell transformation and cancer via increasing mRNA translation of growth-promoting proteins [220]. METTL1 promoted the proliferation, migration and invasion of bladder cancer cells by mediating m7G tRNA modification, thus altering expression of target genes like EGFR/EFEMP1 [221]. Members of the tRNA m7G methyltransferase complex, METTL1 and its partner WDR4, promoted progression and metastasis of head and neck squamous cell carcinoma (HNSCC) via tRNA m7G methylation, thereby enhancing the translation of a subset of oncogenic mRNAs, including genes involved in the PI3K/AKT/mTOR signaling pathway (Fig. 3d) [222]. METTL1/WDR4 also promoted nasopharyngeal carcinoma (NPC) cell EMT and chemoresistance to docetaxel and cisplatin by mediating the translation efficiencies of mRNAs in the WNT/β-catenin signaling pathway (Fig. 3d) [223]. METTL1/WDR4 were also reported to promote ESCC progression by methylating tRNA m7G, thereby sustaining translation of a subset of oncogenic transcripts of the RPTOR/ULK1/autophagy axis (Fig. 3d) [224].

NSUN2 and METTL1 expression induced resistance to the chemotherapeutic agent 5-fluorouracil (5-FU) in HeLa cells, whereas combined NSUN2/METTL1 knockdown drastically potentiated 5-FU sensitivity of cells (Fig. 3d) [225]. Phosphorylation of NSUN2 or METTL1 by Aurora-B or Akt, respectively, abolished their tRNA modifying activities [225]. Inactivation of the yeast METTL1 orthologue, Trm10, increased 5-FU sensitivity by decreasing both Ψ and m7G on tRNAs to obtain a cooperative tRNA destabilization [226]. In intrahepatic cholangiocarcinoma (ICC), co-repressing METTL1 and its downstream chemokine pathway inhibited recruitment of myeloid-derived suppressor cells (MDSCs) and improved anti-PD-1 efficacy [227]. METTL1/WDR4-mediated tRNA m7G promoted HCC resistance to the tyrosine kinase inhibitor Lenvatinib [228].

##### WBSCR22/BUD23

**Fig. 4:**
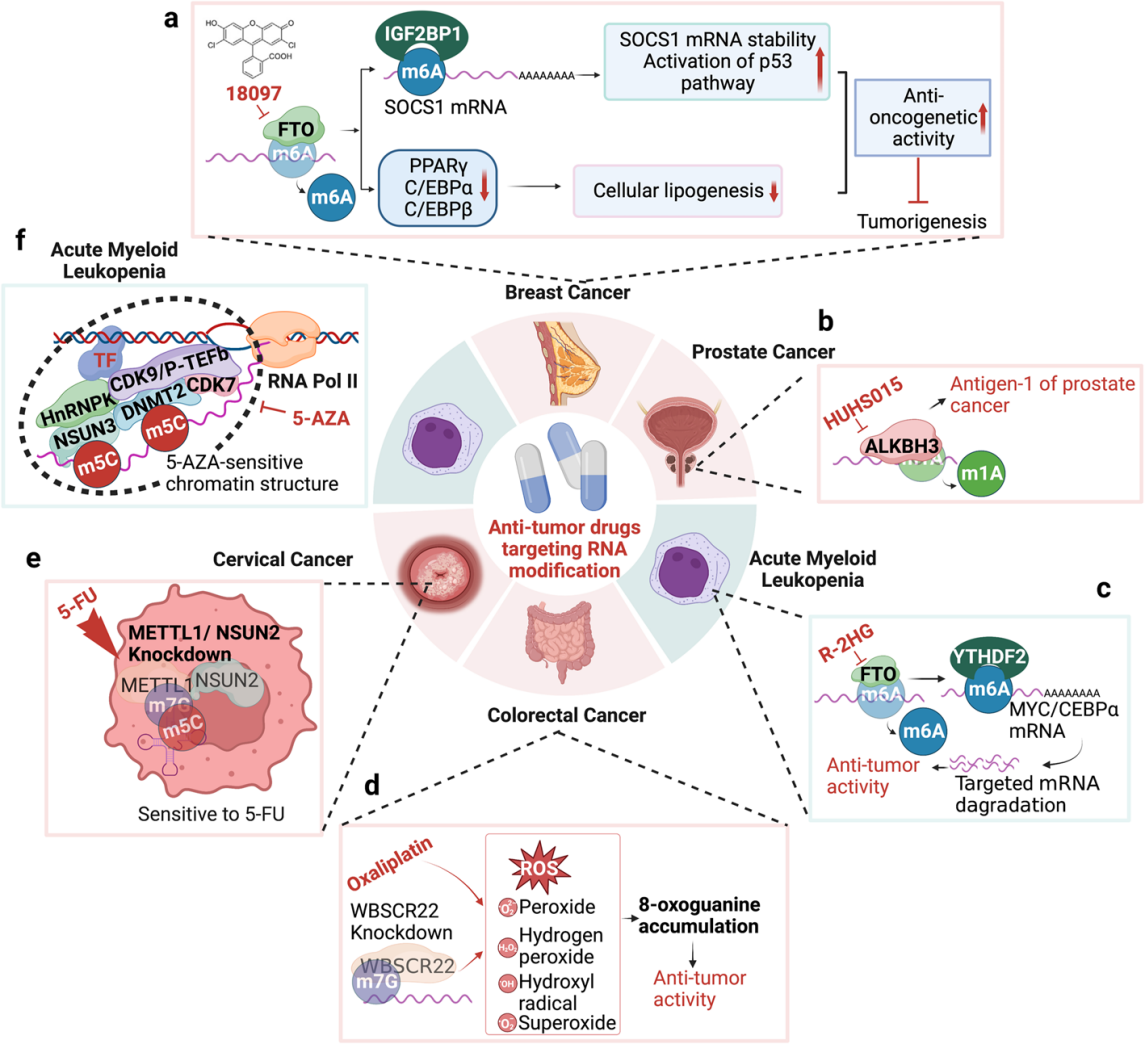
Detailed mechanisms of anti-tumor drugs targeting RNA modifications. **a** 18097 inhibits FTO, thus increasing m6A modification on substrate mRNAs in breast cancer. **b** HUHS015 disturbs the function of ALKBH3, which serves as a prostate cancer antigen. **c** R-2HG prevents FTO removal of m6A modification from MYC/CEBPα in AML. **d** WBSCR22 knockdown enhances the sensitivity of colorectal cancer cells to oxaliplatin. **e** METTL1/NSUN2 knockdown sensitizes cervical cancer cells to 5-FU treatment. **f** NSUN3/DNMT2/CDK7/HnRNPK/CDK9/p-TEFb complex binds nascent nuclear RNA, forms a 5-AZA-sensitive chromatin structure in AML

By analyzing the TCGA cohort, Yan et al. found significantly elevated expression of the rRNA m7G methyltransferase WBSCR22 in human CRC tissue, which led to oxaliplatin resistance [229]. Silencing WBSCR22 sensitized cells for oxaliplatin treatment by increasing the intracellular ROS production induced by oxaliplatin and the 8-oxoguanine oxidative lesion accumulation induced by ROS [229] (Fig. 4d). Using bioinformatic analysis, Chi and colleagues determined that WBSCR22 was overexpressed in glioma tissues and predicted an unfavorable patient prognosis [230]. WBSCR22 loss inhibited glioma cell growth, invasion and migration by reducing Akt/GSK3β phosphorylation and decreasing β-catenin/CyclinD1 levels [230].

WBSCR22 and its cofactor TRMT112 synergistically suppressed tumorigenesis and progression of pancreatic cancer by transcriptionally regulating interferon‑stimulated gene 15 (ISG15), a ubiquitin‑like modifier enzyme involved in metabolism and proteasome degradation [129].

##### RNMT

RNA methyltransferase (RNMT) recruitment to the promoters of genes in the canonical Wnt/β-catenin signaling pathway promoted by MYC-mediated Ser 5 phosphorylation of RNA Polymerase II enhanced mRNA cap methylation and increased translational capacity and elevating protein expression in cancer cells (Fig. 3d) [231].

In summary, the aberrant regulation of RNA methylation is associated with cancer development [232]. The four well-studied RNA methylation modifications including m6A, m1A, m5C, and m7G are involved in poor prognosis and immune microenvironment in multiple tumors such as HCC [19, 233], cervical cancer [234], osteosarcoma [235], pancreatic cancer [91], and breast cancer [236]. The RNA transcripts enriched with these four methylation modifications, especially lncRNAs, may be useful biomarkers for early diagnosis [237] and for estimating cancer patient prognosis.

#### Ψ dysregulation in cancer

Pseudouridine (Ψ) is one of the most abundant modifications found in various RNA species, including mRNA, tRNA, rRNA, and ncRNAs [190]. Ψ plays essential roles in RNA structure, stability, and function. While its specific implications in cancer are not yet fully elucidated, it may potentially regulate the expression of genes involved in cancer-related pathways by enhancing the stability of specific RNA transcripts, altering local and global RNA structures, influencing the splicing machinery, modulating the binding affinity and specificity of RNA-binding proteins, as well as affecting ribosome structure and function. In a study assessing urinary excretion of Ψ in patients with Hodgkin’s disease or non-Hodgkin’s lymphoma, the level of Ψ excretion correlated with clinical stage in high-grade malignant (HGM) lymphoma [238]. Ψ may also be predictive for prostate cancer since its levels are higher androgen-independent cells than in androgen-sensitive or in immortalized human prostate cells [239].

##### Pseudouridine Synthase (PUS) family

One of the best characterized PUS1 targets is the steroid receptor RNA activator 1 (SRA1) ncRNA [240]. PUS1-mediated modification was essential for SRA1 interaction with nuclear receptors, such as oestrogen receptors in breast cancer cells and with retinoic acid receptor-γ (RARγ) in melanoma cells [240]. PUS10 regulated apoptosis induced by TNF-related apoptosis-inducing ligand (TRAIL) in p53-deficient prostate cancer cells [241]. Cells depleted with PUS10 were protected from apoptosis, although whether this effect was dependent on the catalytic activity of PUS10 and direct PUS10 targets is still unclear [6].

##### Dyskerin Pseudouridine Synthase 1 (DKC1)

DKC1, a member of a snoRNP complex, contains TruB Ψ synthase motifs and requires an RNA guide for its catalytic activity on rRNA, snRNA, snoRNA and TERC [242]. DKC1 plays a complex role in cancer and exhibits both oncogenic and tumor-suppressive functions. The dual nature of DKC1's roles can be attributed to its involvement in multiple cellular processes and the context-specific effects it exerts. DKC1-encoded dyskerin was associated with the formation of certain small RNAs and the telomerase activity [243]. Telomerase prevents telomere shortening during cell division and promotes cellular immortality. In this context, the role of DKC1 in telomerase function may contribute to its oncogenic potential by enabling the immortalization of cancer cells and promoting cell proliferation. DKC1 is also involved in ribosome biogenesis. Altered ribosome biogenesis can affect protein synthesis rates, cellular homeostasis, and cell growth. Dysregulation of DKC1 in certain cancer types leads to aberrant ribosome biogenesis and impaired protein synthesis, resulting in a tumor-suppressive effect by impairing cell proliferation and promoting cell cycle arrest. For example, DKC1 overexpression in prostate cancer was necessary for extensive tumor growth, possibly due to its critical function in sustaining protein biosynthesis [244].

DKC1 overexpression in HCC patients was correlated with MYC and MKI67 expression, thus may be an unfavorable prognostic factor predicting advanced clinical stage and poor patient prognosis [245]. In lung cancer patients, the correlation of DKC1 with poor prognosis was linked to its role in maintaining high levels of TERC [246]. DKC1 expression was elevated in glioma tissues and was linked to the WHO stages of tumors [243]. Knockdown of DKC1 significantly inhibited glioma cell growth and motility, possibly by inhibiting the expression of N-cadherin, HIF-1α, and MMP2 [243].

Mutations inactivating DKC1 led to X-linked dyskeratosis congenita, a rare bone-marrow failure disorder that predisposes patients for cancer [242]. DKC1-deficient mice showed decreased pseudouridylation of 28S rRNA, resulting in dysfunctional translation of key mRNAs encoding tumor-related proteins such as VEGF and eventually dyskeratosis congenita-associated phenotypes [247]. Mutations in *TP53* gene were responsible for the inactivation of p53 function as tumor suppressor in over half of human cancers [248]. In breast cancer, DKC1 knockdown led to decreased p53 mRNA translation due to a specific impairment of IRES-mediated translation initiation, thus decreasing p53 protein level and functional activity [248]. Loss of DKC1 function impaird IRES-mediated p27 translation and contributes to spontaneous pituitary tumorigenesis [249].

Taken together, DKC1 seemed to affect cancer cells in two opposite ways. On one hand, DKC1 depletion in dyskeratosis congenita promoted cancer development through dysregulated translation, whereas on the other hand, elevated DKC1 expression could promote telomerase activity in some cancers [6]. However, the second mechanism lacks consistent validation compared to the first, further studies are thus necessary to determine whether DKC1 plays a oncogenic role [6].

#### A-to-I editing dysregulation in cancer

ADAR-catylized A-to-I editing is particularly prevalent in dsRNA regions, including repetitive elements, ncRNAs, and certain mRNA sequences. Dysregulation of A-to-I editing has been observed in multiple cancers. Altered ADAR expression or activity leads to abnormal editing patterns, which impact cancer-related processes. Aberrant editing can result in changes in protein function, deregulation of key signaling pathways, and disrupted RNA regulatory networks. Additionally, abnormal editing events in non-coding regions can influence the expression of oncogenic or tumor-suppressive transcripts, contributing to cancer development and progression.

##### ADAR1

ADAR1 overexpression has been reported in multiple cancer types, including HCC and Chronic myelogenous leukemia (CML) [250]. In HCC, ADAR1-mediated A-to-I mRNA editing on antizyme inhibitor 1 (AZIN1) led to a serine-to-glycine substitution in AZIN1 that induced a cytoplasmic-to-nuclear translocation and neutralized antizyme-mediated degradation of cyclin D1 (CCND1) and ornithine decarboxylase (ODC), eventually promoting tumorigenesis and aggressive behavior (Fig. 3e) [251]. A similar mechanism has also been described in gastric cancer as well as colorectal cancer [252, 253].

Another mechanism that accounts for the oncogenic role of ADAR1 is its regulation of the processing or target specificity of miRNAs. ADAR1 promoted leukemia stem cell (LSC) self-renewal capacity through let-7 pri-microRNA editing and LIN28B upregulation [254]. Bladder cancer-associated (BLCAP) inhibited STAT3 phosphorylation, whereas A-to-I RNA editing by ADAR1 suppressed this inhibition to STAT3 activation in cervical cancer cell lines, thus driving the progression of cervical carcinogenesis [255]. ADAR1-mediated Alu-dependent RNA editing of glioma-associated oncogene (GLI1), a transcriptional activator of the Hedgehog pathway, promoted immunomodulatory drug resistance in multiple myeloma [256]. ADAR1 promoted migration and invasion of lung adenocarcinoma by editing a specific intronic site at the 3′ UTR of focal adhesion kinase (FAK) mRNA [257]. In breast cancer, ADAR1 sustained cell viability and conferred methotrexate resistance in MCF-7 cells through miR-125a-3p/miR-25-3p-dependent A-to-I RNA editing of dihydrofolate reductase (DHFR) mRNA (Fig. 3e) [258]. A major role of human ADAR1 is to inhibit dsRNAs generated from the Alu repeats and PKR hyperactivation, thereby preventing activation of the interferon response and suppressing innate immunity [259, 260]. Decreased ADAR1 activity resulted in dsRNA accumulation, MDA5 (dsRNA sensor)-dependent spontaneous interferon production and PKR activation, thus inducing apoptosis and growth arrest [259]. Therefore, high ADAR1 levels generally suppress the immune response in cancer cells. Recently, loss of ADAR1 function sensitized tumor cells to immunotherapy by reducing A-to-I editing of interferon-inducible RNA species and increasing dsRNA sensing by MDA5 and PKR, which led to growth inhibition and tumor inflammation [261].

In a few studies, ADAR1 was also identified as a tumor suppressor. A-to-I RNA-edited GABRA3 by ADAR1p110 restrained breast cancer cell invasion and metastasis by inhibiting GABRA3-mediated AKT activation (Fig. 3e) [262]. Shoshan et al. also showed ADAR1 downregulation in metastatic melanoma cell lines and tumor specimens, which was contrary to the conclusions from the study by Ishizuka et al. also conducted in melanoma cells [261, 263]. Re-expressing ADAR1 led to inhibition of melanoma proliferation and metastasis through adenosine-to-inosine editing in miR-455-5p (Fig. 3e) [263]. Making the issue even more complicate, it has been reported that peptides derived from A-to-I edited mRNAs could be processed as cancer antigens to elicit immune responses against melanoma cells *in vivo* [264].

In summary, ADAR1 played distinct roles in cancer by editing mRNA, dsRNA and miRNAs, among which ADAR1 function in dsRNA-associated interferon response suppression and immune cell activation seemed to be the main *in vivo* mechanism [6].

##### ADAR2

Compared to ADAR1, ADAR2 is generally considered a tumor suppressor, especially in aggressive brain tumors [265]. Decreased ADAR2 editing activity correlated with higher grade of pediatric astrocytoma, whereas ADAR1/3 was a highly expressed in tumors compared to para-cancerous normal tissues [266]. Reintroducing ADAR2 editing status led to a considerably decreased in proliferation, cell cycle, and migration. Elevated levels of ADAR1 in astrocytoma interfered with ADAR2 specific editing activity by forming ADAR1/2 heterodimers [266]. High-grade astrocytomas generally displayed a significant loss of ADAR2-mediated RNA editing activity [267]. Surprisingly, Tomaselli and colleagues found a considerable rescue of ADAR2 editing activity at relapse in a peculiar patient showing prolonged survival, indicating that ADAR2 might be a possible biomarker predicting long-term survival in high-grade astrocytoma patients [267]. Indeed, ADAR2-mediated A-to-I RNA editing is impaired in glioblastoma and astrocytoma cell lines [265]. Rescue of ADAR2 editing activities in astrocytoma prevented tumor growth by modulating cell division cycle 14B (CDC14B) pre-mRNA editing and in turn influencing downstream S phase kinase-associated protein 2 (SKP2)/p21/p27 axis (Fig. 3e) [265]. ADAR2 was also responsible for upregulating p27 by downregulating the expression of the p27-targeting onco-miRNAs, such as miR-221/222/21, via editing their precursors (Fig. 3e) [268]. In normal brain cells, mir-589-3p edited by ADAR2 inhibited glioblastoma cell proliferation, migration and invasion by retargeting miR-589-3p from the tumor-suppressor protocadherin 9 (PCDH9) mRNA to the mRNA encoding the metalloproteinase 12 (ADAM12) [269]. ADAR2 functions in other cancers are less well characterized. In ESCC cell lines, ADAR2 induced apoptosis and inhibited tumor growth by editing the mRNAs of insulin-like growth factor binding protein 7 (IGFBP7), Filamin B (FLNB) and AZIN1 [270, 271], whereas its editing activity on the mRNA of the membrane transporter solute carrier family 22 member A3 (SLC22A3) drove early tumor invasion and metastasis of familial esophageal cancer in high-risk individuals [272].

##### ADAR3

The third member of the ADAR family, ADAR3, appeared to play a role in glioma cell malignant transformation by mediating cell proliferation, cell adhesion or angiogenesis through manipulating GRIA2Q607R editing level [273].

While A-to-I editing is a crucial RNA modification process, its specific roles and implications in cancer are still being actively investigated. Further research is needed to fully understand the extent of A-to-I editing dysregulation in different cancer types and its functional consequences in cancer biology.

#### ac4C dysregulation in cancer

The functional roles and implications of ac4C in RNA biology, including its relevance to cancer, are not yet well-established or thoroughly explored. N-acetyltransferase 10 (NAT10) is the only currently known enzyme that mediates mRNA ac4C modification and is crucial for mRNA stability and translation efficiency [274]. The LINC00623/NAT10 signaling axis promoted pancreatic ductal adenocarcinoma (PDAC) progression by maintaining the stability of oncogenic mRNAs and promoting their translation efficiency through ac4C modification [275]. By constructing an ac4Cscore model and classifying liver cancer patients into ac4C-high and ac4C-low groups with different prognosis to investigate the potential intrinsic and extrinsic characteristics of tumor, Liu et al. demonstrated that patients subject to the ac4C-high group was related to more aggressive tumor phenotypes, whereas patients attributed to ac4C-low group correlated with less aggressive tumor phenotypes, indicating that ac4Cscore may be a novel biomarker that predicts patient prognosis with anti-PD1 immunotherapy and/or mTOR inhibitor treatment [276]. NAT10 was also overexpressed in CRC and was correlated with shorter patient survival [274]. NAT10 promoted CRC cell proliferation, migration and invasion, as well as tumor formation and metastasis by inhibiting ferroptosis through ac4C modification and stabilization of the ferroptosis suppressor protein 1 (FSP1) transcript (Fig. 3f) [274]. NAT10 promoted ER stress-mediated metastasis and apoptosis resistance to Lenvatinib in HCC cells by increasing the ac4C acetylation level of HSP90AA1 mRNA, maintaining the stability of HSP90AA1, thereby upregulating HSP90AA1 expression (Fig. 3f) [277]. Gastric cancer (GC) showed elevated levels of ac4C mRNA modification as well as its acetyltransferase NAT10, which correlated with disease progression and poor patient prognosis [278]. NAT10 promoted GC cell G2/M phase progression, tumorigenicity and proliferation by mediating ac4C modification and stabilization of MDM2 mRNA transcript, leading to its own upregulation and p53 downregulation (Fig. 3f) [278]. In 2021, Kudrin’s group showed on the BioRxiv preprint that ac4C could be deacetylated by SIRT7 and recognized by the nucleolar protein NOP58 [163]. They suggested that SIRT7 and NOP58 were involved in pre-ribosomal RNA processing and snoRNA function (Fig. 3f) [163]. They also demonstrated that the ac4C level reduction in a NAT10 deficient cell line affected both pre-rRNA processing and snoRNA sub-nuclear localization [163].

Our current knowledge regarding the ac4C modification is still very limited. More high-throughput sequencing analyses along with molecular and cellular validation assays are needed to elucidate further the regulatory mechanism of ac4C as well as its functional consequences in cancer.

#### Summary of RNA modification dysregulation in cancer

While RNA modification study in cancer has made significant progress, challenges and research gaps still need to be addressed. Some RNA modifications have been associated with both tumor-suppressive and oncogenic roles in different contexts. For example, m6A modifications can promote the degradation of oncogenic transcripts or enhance the stability of tumor-suppressive transcripts. The precise effects of specific RNA modifications on cancer development and progression can vary depending on the cellular context and the specific RNA species involved. It is thus crucial to investigate the specific roles and consequences of RNA modifications within the context of individual cancer types to gain a more comprehensive understanding of their functional significance. Besides, regardless of the growing evidence linking specific RNA modifications to cancer development and progression, correlation does not always imply causation, further functional studies are thus needed to determine whether RNA modifications directly contribute to tumorigenesis or if they are simply associated with other underlying molecular changes in cancer cells.

RNA modifications do not act in isolation but interact with other regulatory mechanisms, such as alternative splicing, noncoding RNAs, and RNA binding proteins. Investigating the interplay between RNA modifications and these regulatory layers is essential to understand their coordinated roles in cancer progression and to develop more effective therapeutic drugs. For example, Okamoto et al.showed that combined NSUN2/METTL1 knockdown sensitized HeLa cells to 5-FU treatment, suggesting that interfering with tRNA methylation may be a promising rationale to improve 5-FU chemotherapy in cancer treatment (Fig. 4e) [225]. Moreover, integrating RNA modification data with other omics data, such as gene expression profiles, genomic alterations, and epigenetic modifications, will provide a more comprehensive understanding of the molecular landscape of cancer. Developing computational methods and analytical tools to effectively integrate and interpret these multi-omics datasets will facilitate the discovery of new insights into cancer biology.

RNA modifications have the potential to serve as biomarkers for cancer diagnosis, prognosis, and treatment response. Identifying and validating specific modification patterns or signatures that correlate with different cancer types or disease states would aid in early detection and personalized treatment strategies. Studying the regulatory mechanisms that control RNA modifications in each specific cancer type and disease stage will provide valuable insights into the underlying molecular processes. Further exploring the roles of RNA modifiers in cancer cells may uncover novel therapeutic targets and inform the development of targeted interventions. Elucidating the dynamics of RNA modifications in cancer is an important research direction. Investigating how modifications change in response to therapeutic interventions, disease progression, or specific cellular contexts will provide insights into their functional roles and potential as therapeutic targets. Developing strategies to target and manipulate RNA modifications for therapeutic purposes is also an exciting area of research. This includes the development of small molecules, antibodies, or gene editing tools to modulate specific modifications and investigate their therapeutic potential in cancer treatment. Exploring the feasibility and implications of RNA modification editing, similar to DNA base editing, is also an important emerging area of interest. Developing precise editing tools to manipulate specific RNA modifications in cancer cells would enable the exploration of their functional consequences and therapeutic potential.

Addressing these gaps will require collaborative efforts among researchers, technological advancements, larger and well-characterized patient cohorts, and multidisciplinary approaches. As research progresses, we will gain deeper insights into the roles of RNA modifications in cancer and potentially uncover novel therapeutic targets and biomarkers for improved cancer diagnosis and treatment.

### Neurological disorders

Neurological disorders are a diverse group of conditions that affect the structure and function of the nervous system, including the brain, spinal cord and peripheral nerves. Dysregulation of RNA modifications are involved in multiple neurological disorders, such as dysregulated brain development, stroke, neurodegenerative diseases and traumatic injuries.

#### m6A dysregulation in neurological disorders

Among all organs, m6A methylation of RNAs is highest in the brain and is known to promote cell survival after adverse conditions [279].

In embryonic mouse brains, Mettl14/Mettl3 knockout-induced m6A modification defect prolonged cell cycle of radial glia cells and extended cortical neurogenesis into postnatal stages [128]. m6A modification promoted the decay of mRNAs enriched in embryonic mouse cortex, including transcripts related to transcription factors, cell cycle, neurogenesis and neural differentiation [128]. m6A signaling also regulated human cortical neurogenesis in forebrain organoids [128]. Defects in m6A demethylases have also been linked to neurological defects [22]. Dysregulation of the m6A pathway led to axonal overgrowth and misguidance and was therefore associated with neurodevelopmental defects and neural dysfunctions [280]. Ythdf, the main m6A reader in *Drosophila* nervous system, inhibited translation of key transcripts involved in axonal growth regulation via its interaction with Fmr1, the fly homolog of Fragile X mental retardation RNA binding protein (FMRP) [280]. YTHDF1 regulated axon guidance by modulating the translation of axon guidance receptor Robo3.1 [281]. A novel m6A reader, Proline rich coiled-coil 2A (Prrc2a) controlled oligodendrocyte specification and myelination by recognition of a consensus GGACU motif in the CDS region of Olig2 in an m6A-dependent manner, thus stabilizing Olig2 mRNA [123]. Interestingly, this m6A modification of Olig2 mRNA could be erased by Fto, leading to Olig2 mRNA degradation [123]. Environmental factors, such as cobalt exposure, can also cause memory impairment and cognitive deficits by affecting m6A modification [282]. CoCl_2_ exposure mediated the expression and enzymatic activity of m6A modifying enzymes in C57BL/6 mouse cortex and human neuroblastoma H4 cells, resulting in differentially m6A-modified and translated genes enriched in synaptic transmission and central nervous system (CNS) development related pathways [282].

RNA m6A also plays an essential role in brain development and controls translation of important genes involved in pathways associated with Alzheimer’s disease (AD) [116]. A recent study revealed a m6A methylation decrease in brains of aged mice and AD patients compared to young mice and cognitively intact human subjects, respectively, in transcripts related to synaptic function, including AMPA-selective glutamate receptor 1 (Glua1) and calcium/calmodulin-dependent protein kinase 2 (CAMKII), resulting in decreased synthesis of synaptic proteins, such as GLUA1 and CAMKII [283]. Genetic variation in Introns 1&2 of the FTO gene may predict risk of AD [284]. Down-regulation of m6A RNA methylation by FTO induced N-methyl-D-aspartate (NMDA) receptor 1 expression, elevating Ca^2+^ influx and oxidative stress, resulting in dopaminergic neuron apoptosis [285].

In the stroke model, m6A modification may play protective or harmful roles. On the one hand, in the early stage of acute ischemic stroke, METTL3-mediated m6A methylation also enhanced miR-335 maturation, promoting stress granule formation and inhibiting apoptosis of injury neurons [286]. Hypothermia protected neurons from neuronal ischemia/reperfusion-induced pyroptosis through m6A-mediated activation of phosphatase and tensin homologous protein (PTEN) and the phosphatidylinositol-4,5-bisphosphate 3-kinase (PI3K) / protein kinase B (Akt) / glycogen synthase kinase-3β (GSK-3β) signaling pathway [287]. m6A demethylases Alkbh5/Fto protect neurons from damage after cerebral ischemia–reperfusion injury by selectively demethylating the Bcl2 mRNA, preventing Bcl2 mRNA degradation and thus enhancing Bcl2 translation [288]. YTHDC1 facilitated neuronal survival after ischemic stroke by promoting PTEN mRNA degradation, thereby increasing Akt phosphorylation [289]. On the other hand, mice subject to transient middle cerebral artery occlusion showed significantly increased global m6A levels by markedly decreased FTO levels [279]. Oxygen glucose deprivation/re-oxygenation (OGD/R) induced neuronal cell apoptosis by downregulating the expression of lncRNA D63785 (Lnc-D63785) through increased METTL3-mediated Lnc-D63785 m6A modification, thereby inducing miR-422a accumulation and leading to downregulation of miR-422a targets mitogen-activated protein kinase kinase 6 (MAPKK6) and myocyte enhancer factor-2D (MEF2D) [290].

Traumatic brain injury (TBI) induced METTL3 downregulation, leading to 922 differentially expressed m6A-modified mRNA transcripts in mouse hippocampus, suggesting that m6A modification changes in the early TBI period may be promising therapeutic targets [291]. METTL14 and FTO expression was also remarkably down-regulated in cerebral cortex in response to TBI. Functional FTO was essential to repair TBI-induced neurological damage [292]. In zebrafish, spinal cord injury (SCI) induced an epitranscriptomic change, altering Mettl3 transcription level as well as m6A RNA methylation and transcription levels of genes associated with neural regeneration [125]. Moreover, the expression of METTL3 was increased in both astrocytes and neural stem cells, indicating that m6A RNA methylation may contribute to spinal cord regeneration [125]. METTL14-mediated m6A modification inhibited Ras-related dexamethasone-induced 1 (RASD1) and induced neuron apoptosis in SCI by promoting the maturation of pri-miR-375 to miR-375 [293]. Depletion of either m6A related factors, METTL14 or YTHDF1, diminished global protein translation induced by sciatic nerve lesion in adult dorsal root ganglion (DRG) and reduced functional axon regeneration in the peripheral nervous system [294]. Moreover, in the adult CNS, METTL14 loss also attenuated axon regeneration of retinal ganglion neurons induced by PTEN deletion [294].

Although much is known about m6A modification, our knowledge regarding its relevance in neurological disorders is still in an early stage. Based on our current knowledge, m6A-associated modifiers hold great potential in therapeutic treatment of neurological disorders. However, further study and maybe much cooperation are needed for a deeper and more thorough understanding of m6A functions in neurological disorders.

#### A-to-I editing dysregulation in neurological disorders

A-to-I editing plays essential role in the CNS and has been implicated in various neurological disorders. It can impact ion channel functions, which play a critical role in neuronal excitability and signaling. It also affects the neurotransmitter receptors and transporters crucial for synaptic transmission and neuronal communication. Furthermore, A-to-I editing modifies RNA secondary structure, affecting RNA–protein interactions and splicing processes. Altered editing levels in non-coding regions, such as 5' and 3' UTRs or introns, can impact RNA stability, localization, or alternative splicing patterns. Editing in non-coding RNAs may impact their binding affinity and target specificity, thus altering gene expression regulation.

The A-to-I editing at the GluR2 Q/R site regulates tetramerization of α-amino-3-hydroxy-5-methyl-4-isoxazole propionate (AMPA)-type glutamate receptors (AMPARs), which play a significant role in excitatory synaptic transmission and plasticity [295]. Specifically, subunits with unedited Q tetramerize readily trafficked to synapses, whereas subunits with edited R were mostly unassembled and retained in ER, thus limiting the amount of the functionally critical R subunits within AMPAR tetramers [295].

In early development, splicing and editing AMPAR transcripts were important for activity-dependent dendritic growth in a cell-class-specific manner [296]. ADAR2-mediated A-to-I editing in the pre-mRNA of the AMPAR subunit GluA2 is critical for survival, at least during the first few weeks of life [297, 298]. Transcripts encoding the α3 subunit of heteromeric GABAA receptors (Gabra3, a part of the major inhibitory neurotransmitter system in the CNS), are often edited at the I/M site, where isoleucine (ATA) is converted to methionine (ATI) in a region that encodes the predicted third transmembrane domain [299]. During brain development, upregulation of the nonedited α3(I)β3γ2L GABAA receptors may allow the robust excitatory responses that are important for normal synapse formation [299]. ADAR-mediated A-to-I RNA editing in genes encoding for the neuron-specific RNA binding proteins human antigen B/D (HuB/D) is remarkably crucial for mammalian brain development [300]. Evolutionarily conserved A-to-I editing increased protein stability of the brain-specific alternative splicing factor Nova1 [301]. FLNA and CYFIP2 are also evolutionarily conserved human A-to-I RNA editing targets that play important roles in proper nervous system function [302]. A-to-I editing of microRNAs increases during mammalian brain development [303].

A-to-I conversion in GluA2 mRNA led to a Q-to-R substitution in GluA2 protein, which regulated the Ca^2+^-permeability of the AMPAR [16]. A defect in the editing of the mRNA transcript led to an unedited Q/R site in the GluA2 subunit of glutamate AMPAR in the spinal motor neurons of patients with ALS, interfering with the correct functioning of the glutamate receptors and may thereby cause neuronal death in ALS patients [16, 304]. ADAR2-negative motor neurons in patients with sporadic ALS had cytoplasmic inclusions that were immunoreactive to phosphorylated TDP-43 but lacked non-phosphorylated TDP-43 in the nucleus [305]. In addition, conditional ADAR2 knockout mice showed increased autophagy in their spinal motor neurons [306], inconsistent with the results observed in ALS individuals [22]. Human intravenous injection of adeno-associated virus serotype 9 (AAV9)-ADAR2 in conditional ADAR2 knockout mice (AR2), which comprise a mechanistic sporadic ALS mouse model, rescued the motor neurons of AR2 mice from death by normalizing TDP-43 expression [307]. ADAR2 deficiency can occur in ALS patients with a FUSP525L mutation in the fused in sarcoma (FUS) gene and is unrelated to the presence of FUS-positive cytoplasmic inclusions in motor neurons, suggesting that FUS-associated ALS may share neurodegenerative characteristics with classical sporadic ALS [308]. A-to-I RNA editing at a novel site in intron 7 of EAAT2 pre-mRNA was significantly higher in spinal cord and motor cortex from ALS patients compared to that in cerebellum and appeared to activate a cryptic alternative polyadenylation site [309]. Mutations in ADAR1, IFIH1, and RNASEH2B led to a spastic paraplegia phenotype in patients [310]. By assessing the A-to-I mRNA editing levels in cortex samples of 20 subjects 22–102 years old, Nicholas et al.demonstrated that CYFIP2 (implicated in synaptic maintenance) mRNA editing level significantly decreased in an age-dependent manner, whereas GABRA3 editing remained much more stable with age, indicating a gene-specific age-related RNA editing decline [311]. Loss of all ADAR2-mediated edits led to significant changes in transcript profiles, hearing, behavior and allergy parameters of brain [297]. Increased 5HT2CR pre-RNA editing in Prader-Willi syndrome (PWS) mice was associated with alterations in 5HT2CR-related behaviors, including locomotor activity, impulsive responding and reactivity to palatable foodstuffs, while no obvious effect was seen in non-5HT2CR-related behaviors such as marble burying [312].

Besides neuronal development and motor neuron functions, dysregulation of A-to-I editing has also been reported in other neurological disorders. For example, ADAR2-mediated editing at Q/R site of GluA2 determined vulnerability of neurons in the rat hippocampus to forebrain ischemia [313]. SCI strongly reduced the editing at the R/G site of GluRs of AMPAR and reduced post-synaptic excitatory response to glutamate, thus limiting the progression of cell death [314]. Mice deficient in GluR6 Q/R site editing showed induced NMDA receptor-independent long-term potentiation (LTP) at the medial perforant path-dentate gyrus synapse and thus were more vulnerable to kainite-induced seizures [315].

#### Dysregulation of other RNA modifications in neurological disorders

##### NSUN2 (m5C)

The cytosine-5 RNA methyltransferase NSUN2 is highly expressed in early neuroepithelial progenitors and is gradually reduced during human neuroepithelial stem (NES) cell differentiation [156]. Loss-of-function mutations in NSUN2 caused neurodevelopmental disorders in humans by increasing angiogenin-mediated endonucleolytic cleavage of tRNA [156]. NSUN2 repression also inhibited migration of neural cells toward the chemoattractant fibroblast growth factor 2 (FGF-2), which might be a contributing factor for the impaired differentiation capacity [156]. In NSun2-mutant patient fibroblasts and NSun2-deficient mice, tRNAs lacking NSun2-mediated methylation were bound by angiogenin with higher affinity, leading to increased endonucleolytic cleavage of tRNA [15]. Accumulation of 5’ tRNA-derived small RNA fragments attenuated protein translation and triggered cellular stress responses, resulting in decreased cell size and increased neuronal apoptosis [15]. Deletion of *Drosophila melanogaster* NSUN2 ortholog, CG6133, led to severe short-term-memory (STM) deficits, which could be rescued by wild-type NSUN2 re-expression in the nervous system [14].

##### TRMT family (m1A)

Compared with the wild type, the 5XFAD AD mice displayed hypo-m1A-methylation in both mitochondrial (methylated by TRMT10C and HSD17B10) and cytosolic tRNAs (methylated by TRMT61A), knockdown of which resulted in a more severe phenotype in Drosophila [316].

#### Summary of RNA modification dysregulation in neurological disorders

The field of RNA modification study in neurological disorders is a dynamic area of research with ongoing investigations and emerging findings. While significant progress has been made, there are still areas of debate as well as gaps that need to be addressed in this field.

The brain is a complex and heterogeneous organ composed of diverse cell types, each with unique transcriptomic profiles and regulatory processes. It is highly possible that RNA modifications exhibit cell type-specific patterns and functions, which may be overlooked since our current knowledge of RNA modifications in neurological disorders often relies on bulk tissue analyses. The detection and quantification of RNA modifications in the context of neurological disorders pose technical challenges. Existing methods for RNA modification analysis often rely on enrichment or sequencing techniques, which can introduce biases, false positives, or limitations in sensitivity. Variations in sample sizes and heterogeneity of patient populations may also lead to different observations and thus influence our understanding. Advancements in single-cell RNA sequencing and spatial transcriptomics technologies are needed to uncover the cell type-specific landscape of RNA modifications in neurological disorders.

Many neurological disorders are characterized by long-term disease progression and chronic neurodegeneration. However, our understanding of how RNA modifications change over time and contribute to disease progression is limited. Correlation does not always imply causation; determining whether alterations in RNA modifications directly contribute to neurological disorders or are consequences associated with the disease process is still challenging. Understanding how modifications change during critical stages of neural development, in response to environmental stimuli, or during disease progression may reveal important regulatory mechanisms and potential therapeutic windows. Longitudinal studies investigating the dynamics of RNA modifications throughout disease stages and their association with clinical features and outcomes will help elucidate their temporal roles and potential as biomarkers. Developing targeted therapeutic approaches to modulate specific RNA modifications in neurological disorders is an exciting avenue of research. This may involve the development of small molecules, antisense oligonucleotides, or RNA editing technologies to restore normal RNA modification patterns and rescue disease-associated phenotypes. Despite the progress in understanding RNA modifications in neurological disorders, translating this knowledge into effective therapeutic interventions remains a challenge. Investigating specific RNA modifications that are dysregulated in neurological disorders can provide insights into their role in disease pathogenesis.

As research progresses and new technologies emerge, further exploration of RNA modifications in the context of brain function and neurological diseases will likely uncover novel insights and pave the way for innovative therapeutic interventions. Collaboration, replication studies, and multidisciplinary investigations will contribute to resolving these discrepancies and advancing our understanding of the role of RNA modifications in neurological disorders.

### Cardiovascular diseases

Cardiovascular diseases are a group of conditions that affect the heart and blood vessels. They encompass a wide range of disorders, including diseases of the heart, blood vessels, and conditions that can lead to impaired cardiovascular function. Dysregulation of RNA modifications has been reported in multiple cardiovascular diseases, including heart failure, stroke, coronary artery disease and hypertension.

#### m6A dysregulation in cardiovascular diseases

m6A mRNA methylation was increased in human cardiomyopathy and regulated cardiac gene expression and cellular growth [317]. METTL3-mediated m6A methylation was enhanced in response to hypertrophic stimuli and was essential for a normal cardiomyocyte hypertrophic response [25]. Dorn et al. recently reported that the normal hypertrophic response in cardiomyocytes required METTL3-mediated m6A mRNA methylation, which could be induced by hypertrophic stimuli. Increased m6A levels led to compensated cardiac hypertrophy, whereas decreased m6A resulted in eccentric cardiomyocyte remodeling and dysfunction, indicating the significance of this stress-response mechanism in sustaining normal cardiac function [25, 318]. Cardiac-hypertrophy-associated PIWI-interacting RNA (CHAPIR) promoted pathological hypertrophy and cardiac remodeling by controlling METTL3-dependent m6A methylation of Parp10 mRNA [319]. m6A RNA methylation changes contributed to heart failure progression through transcription-independent modulation of translation, where Fto-knockout mice exhibited an impaired cardiac function compared to control mice [320].

The human CD34 + stem cell-derived exosomes played important roles in cardiovascular repair by regulating the mRNA m6A methylation in the ischemic myocardium [318, 321]. METTL3 and ALKBH5 oppositely regulated m6A modification of the master regulator of lysosomal biogenesis and autophagy genes, TFEB, which dictated the fate of hypoxia/reoxygenation-treated cardiomyocytes [26]. Moreover, m6A regulated cardiomyocyte Ca^2+^ dynamics and cardiac function in the ischemic heart, where the key m6A demethylase FTO was significantly downregulated [321]. FTO plays a critical role in cardiac contractile function by selectively demethylating cardiac contractile transcripts to prevent their degradation and to improve their protein expression under ischemia [322].

YTHDF3 variant rs4739066 showed a weak association with myocardial infarction (MI) in a genome-wide association study in Saudis of Arab descent [323]. METTL14 regulated indoxyl sulfate-induced vascular calcification by selectively methylating vascular osteogenic transcripts, thus facilitating their degradation and increasing their translation induced by indoxyl sulfate [324].

Recent studies have demonstrated that dysregulation of circular RNAs (circRNAs) is associated with hypertension and may be used as novel biomarkers and potential therapeutic targets for various forms of hypertension [318]. Su et al.mapped transcriptome-wide m6A circRNAs in hypoxic mediated pulmonary hypertension (HPH), demonstrating m6A influence of in HPH circRNA-miRNA-mRNA coexpression network [325]. Mo et al.demonstrated important roles of m6A-associated single-nucleotide polymorphisms (m6A-SNPs) in blood pressure regulation [326]. FTO genetic variant rs9939609 A/T is positively associated with body mass index (BMI) in women, but negatively associated with diastolic and mean blood pressure in men with hypertension [327]. Both m6A levels and YTHDF1 protein expression were elevated in human and rodent pulmonary hypertension (PH) samples as well as in hypoxic pulmonary artery smooth muscle cells (PASMCs) [328]. YTHDF1 regulated PASMC proliferation and PH development by promoting translation of melanoma-associated antigen D1 (MAGED1) in an m6A-dependent manner [328].

#### Dysregulation of other RNA modifications in cardiovascular diseases

##### m5C

Deficiency of the tRNA methyltransferase Dnmt2/Trdmt1 in mice led to augmented dissociation of the negatively regulating Rn7sk ncRNA component, thus activating the P-TEFb complex and resulting in cardiac hypertrophy [157].

##### m7G

m7G methyltransferase METTL1 promoted post-ischemic angiogenesis and blood flow recovery via regulating VEGFA mRNA translation in an m7G methylation-dependent manner [329]. Wang et al.also reported that m7Gs were differentially modified in HPH, where m7G modified lncRNAs were significantly upregulated compared with non-m7G lncRNAs [330]. The mitochondrial transmembrane (TMEM) protein TMEM11 inhibits cardiomyocyte proliferation and cardiac repair after myocardial injury via METTL1-mediated m7G methylation of ATF5 mRNA [331].

##### Ψ

Ψ and N-formylmethionine were associated with left ventricular mass index (LVMI), highlighting that mitochondrial-derived metabolites may serve as early biomarkers for left ventricular remodeling and subclinical heart failure [332].

##### A-to-I editing

ADAR-mediated A-to-I RNA editing controlled cathepsin S (CTSS) mRNA translation in atherosclerosis by recruiting the stabilizing RNA-binding protein human antigen R (HuR) to CTSS 3′ UTR, thereby enhancing CTSS mRNA stability and translation [333].

##### Ac4C

Heart-apoptosis-associated PIWI-interacting RNA (HAAPIR) regulated cardiomyocyte death after myocardial infarction by boosting NAT10-mediated ac4C modification of transcription factor EC (Tfec) mRNA [334].

#### Summary of RNA modification dysregulation in cardiovascular diseases

The study of RNA modifications in cardiovascular diseases is an emerging field with great potential for understanding disease mechanisms and developing novel therapeutic strategies. However, our understanding of the RNA modification landscape specifically in cardiovascular cells and disease models is relatively limited. Except for m6A, only one or two studies have reported regarding the relevance of each RNA modification in cardiovascular disease, not to mention the exact mechanisms and causal relationships between each RNA modification and disease pathogenesis. The effects of specific RNA modifications in cardiovascular diseases can be context-dependent. The same RNA modification may have distinct functional consequences in different cell types, disease stages, or disease models. Comprehensive profiling and characterization of RNA modifications in cardiovascular tissues, such as the heart, blood vessels, and endothelial cells, are needed to uncover their roles and functional significance in cardiovascular diseases. Investigating RNA modifications that are dysregulated in specific cardiovascular diseases, such as atherosclerosis, heart failure, or arrhythmias, will provide insights into their role in disease pathogenesis. Identifying disease-specific RNA modification signatures or patterns may serve as diagnostic markers and potential targets for therapeutic intervention. Examining the dynamic changes of RNA modifications during disease progression, in response to therapeutic interventions, or different stages of cardiovascular diseases will provide insights into their functional roles and potential as therapeutic targets.

Few studies focused on the role of ALKBH2 and ALKBH3 in cardiovascular diseases. As crucial factors involved in the DNA damage and repair process, it would be meaningful to investigate the association between ALKBH2/3 and the cardiovascular diseases induced by DNA-damage-mediated cell death in cardiomyocytes and vascular endothelial cells [335]. ALKB homologs have great potential in the drug development of cardiovascular diseases and exploring specific ALKBH1 inhibitors may be a good therapeutic strategy for atherosclerosis or hypertension. RNA modifications hold promise as potential biomarkers for cardiovascular diseases. However, the identification and validation of RNA modification-based biomarkers in clinical settings are still in the early stages. ALKBH5 ALKBH8 agonists may be beneficial for treating myocardial ischemia–reperfusion injury due to their function in the modulation of autophagy and oxidative stress [335]. FTO can demethylate m6A-modified transcripts related to cardiac contraction, including myh6/7, SERCA2a, and RYR2. Thus its agonists may improve myocardial ischemia-induced heart failure [335]. Exploiting RNA modifications as therapeutic targets for cardiovascular diseases is an exciting prospect. However, the development of targeted therapies that specifically modulate RNA modifications poses significant challenges. Further research is needed to understand the therapeutic potential of RNA modifications in cardiovascular diseases, including the design of delivery systems, optimization of specificity and efficacy, and assessment of safety profiles.

### Metabolic diseases

Metabolic diseases are characterized by abnormalities in the metabolism. These diseases can affect various organs and systems in the body, including the liver, pancreas, hormones, and the cardiovascular system. Dysregulation of RNA modifications have been reported in metabolic diseases, such as diabetes mellitus, hypoglycemia and obesity.

#### m6A dysregulation in metabolic diseases

METTL3 inhibited hepatic insulin sensitivity via m6A modification of Fatty acid synthase (Fasn) mRNA and promoted fatty acid synthesis in diabetic mice fed with a high-fat diet [336]. METTL14-dependent m6A mRNA methylation regulated human β-cell biology, including cell-cycle progression, insulin secretion, and the Insulin/IGF1-AKT-PDX1 pathway in type 2 diabetes (T2D) [337]. In T2D patients, high-glucose treatment upregulated FTO mRNA expression, resulting in a decrease in m6A, further inducing mRNA expression of metabolic genes such as Glucose-6-Phosphatase Catalytic Subunit 1 (G6PC), and Diacylglycerol O-Acyltransferase 2 (DGAT2) [338]. FTO rs9939609 (T/A) was significantly related to a higher homeostasis model assessment (HOMA) index and familial history of diabetes [339]. Hypoglycemia mediated the expression of hypothalamic miRNAs related to FTO, AP-1 transcription factor subunit (FOS), and Fos proto-oncogene [340].

Obesity is a serious international health problem that increases the risk of several common diseases [341]. FTO is widely expressed in rodent brains including hypothalamic nuclei linked to food intake regulation [342]. A common variant rs9939609 in the FTO gene predisposed to childhood and adult obesity through an additive association with BMI [341]. Marcadenti et al.also demonstrated that common genetic variants of FTO rs9939609 were positively associated with BMI and neck circumference in women [327]. Dina et al.identified multiple variants of FTO that were strongly associated with childhood obesity and severe adult obesity [23]. Another FTO variant rs8061518 in Intron 3 was associated with decreased risk of obesity and low concentration of leptin [24]. Silencing FTO inhibited adipogenesis of preadipocytes by promoting the m6A-YTHDF2-dependent mRNA decay of crucial cell cycle regulators, Cyclin A2 (CCNA2) and Cyclin Dependent Kinase 2 (CDK2), at the early stage of adipogenesis [343]. Endothelial FTO loss protected from obesity-induced metabolic and vascular dysfunction by increasing AKT phosphorylation in endothelial cells and skeletal muscle and preserving myogenic tone in resistance arteries [344]. Zinc finger protein (Zfp217) promoted adipogenic differentiation through orchestration of transcriptional and post-transcriptional regulation, activating the transcription of m6A demethylase FTO as well as interacting with YTHDF2 to facilitate FTO maintenance at m6A sites on various mRNA [146]. FTO deficiency led to increased m6A levels on ATG5 and ATG7 transcripts, which could be captured by YTHDF2, resulting in mRNA degradation and reduced protein expression of ATG5 and ATG7, ultimately leading to attenuation of autophagosome formation and inhibiting autophagy and adipogenesis [345]. Mitochondrial carrier homolog 2 (MTCH2) promoted adipogenesis in intramuscular preadipocytes through YTHDF1-m6A-dependent mechanism [88]. Loss of m6A on Family with Sequence Similarity 134, Member B (FAM134B) promoted porcine preadipocytes adipogenic differentiation and lipid deposition by preventing YTHDF2 recognition as well as upregulating the expression levels of CCAAT/enhancer-binding protein (C/EBPα) and peroxisome proliferator-activated receptor γ (PPARγ) [346].

#### Dysregulation of other RNA modifications in metabolic diseases

##### m5C

A crucial process for mitochondrial ribosome biogenesis is the recruitment of the m5C RNA methyltransferase, NSUN4, to the large ribosomal subunit through binding to the C-terminus of the mitochondrial transcription termination factor (MTERF) family member, MTERF4 [347]. NSUN4 played a dual function in mitochondrial ribosomal biogenesis, methylating cytosine 911 in 12S rRNA (m5C911) of the small subunit on the one hand and cooperating with MTERF4 to assemble the small and large subunits to form a monosome on the other hand [348]. Mutations in another NSUN family member, NSUN3, resulted in deficient methylation m5C and formylation f5C of mt-tRNA(Met) wobble cytosine in a patient who developed mitochondrial disease symptoms combined with developmental disorders and OXPHOS deficiency in skeletal muscle [349]. Nsun3 also regulated embryonic stem cell (ESC) differentiation by promoting mt-tRNAMet methylation and formylation as well as mitochondrial translation and respiration [350]. NSUN2 was necessary for m5C methylation at positions 48, 49 and 50 of several mitochondrial tRNAs, although it did not show a profound effect on mitochondrial tRNA stability and oxidative phosphorylation in differentiated cells [351].

##### m1A

TRMT10C mutations affected mitochondrial RNase P protein 1 (MRPP1) protein stability and mt-tRNA processing, leading to multiple respiratory chain deficiencies [352].

##### A-to-I editing

Mouse ESCs deficient in the RNA-editing enzyme ADAR1 failed to contribute to liver, thymus, spleen, bone marrow, and blood in adult chimeric mice [353]. Dysregulated A-to-I editing of 5-HT2CR mRNAs resulted in constitutive activation of the sympathetic nervous system, energy dissipation and fat mass loss [354].

##### Ac4C

NAT10 regulated fatty acids metabolism in cancer cells by stabilizing fatty acid metabolic genes such as ACAT1, ACADSB, ACSL1, ACSL3, ACSL4 and ELOLV6 through ac4C RNA acetylation [355].

#### Summary of RNA modification dysregulation in metabolic diseases

RNA modifications exhibit tissue-specific and cell type-specific patterns. Although much progress has been made in the field of RNA modification studies, its relevance in metabolic diseases is still a relatively new and emerging field with potential to uncover new insights into disease mechanisms and identify therapeutic targets. Our knowledge of the full spectrum of RNA modifications and their dynamics in the context of metabolic diseases is limited. Comprehensive profiling and characterization of RNA modifications in relevant tissues, such as adipose tissue, liver, pancreas, and muscle, are needed to uncover their roles and functional significance in metabolic diseases. While multiple studies have identified associations between RNA modifications and metabolic diseases through correlative analyses, it is still challenging to determine whether there is a causal relationship; further functional studies are thus needed to determine whether RNA modifications directly contribute to metabolic disease development or if they are simply markers of underlying metabolic dysregulation.

Metabolic diseases encompass a wide range of conditions, including obesity, diabetes, and metabolic syndrome, with distinct underlying mechanisms and genetic or environmental contributors. The heterogeneity of metabolic diseases may contribute to contradictory findings as studies focus on specific subtypes or patient populations. Therefore, identifying disease-specific RNA modification patterns or signatures and examining the dynamic changes of RNA modifications during disease progression, in response to therapeutic interventions, or different metabolic states will provide insights into their functional roles and potential as therapeutic targets.

### Genetic and developmental disorders

Genetic and developmental diseases encompass a wide range of conditions that arise from genetic mutations, alterations in embryonic development, or a combination of genetic and environmental factors. These diseases affect various systems and organs in the body and may present at birth or later in life. Most serious genetic disorders are caused by genetic mutations, but epigenetic or epitranscriptomic regulation of gene expression may also cause dysregulation in development [22]. The loss of RNA modifying enzymes has been considered as the cause of various developmental syndromes and disorders [165].

#### m5C dysregulation in genetic and developmental disorders

Transcripts of the NOL1/NOP2/sun domain-containing RNA methyltransferases Nsun2-7 were enriched in the developing brain, eye, ear, branchial arches, olfactory epithelium, limb and heart, while Nsun2 and Nsun6 were also enriched in the caudal neural tube and newly formed somites, suggesting that functions of NSUN proteins and RNA methylation may overlap during embryonic development [356]. Nsun3 regulated ESC differentiation by generating 5-methylcytosine in the anti-codon loop of mitochondrial tRNAMet, thus promoting mitochondrial activity [350]. The tRNA aspartic acid methyltransferase 1 (TRDMT1) is significantly associated with spina bifida [357]. Missense mutation c.2035G > A (p.Gly679Arg) in NSUN2, a m5C methyltransferase that functions in spindle assembly during mitosis as well as chromosome segregation, caused autosomal-recessive intellectual disability [7]. Similar phenotypes (intellectual disability and facial dysmorphism) were observed in humans with homozygous NSUN2 mutation, suggesting that NSUN2 plays a crucial role in intellectual disability prevention [14].

The nucleolar protein NSUN1 may play a role in regulating the cell cycle in Cri-du-chat (CDC) syndrome, a chromosomal syndrome resulting from partial deletions of Chromosome 5 [18]. Homozygous splice mutation in NSUN2 caused Dubowitz-like syndrome by abolishing the canonical Exon 6 splice acceptor site and using a cryptic splice donor within an AluY, leading to subsequent mRNA instability [19]. Using whole exome sequencing (WES) approach, Fahiminiya et al. identified a novel homozygous deletion in NSUN2 in a male proband with Noonan-like syndrome [358]. The BUD23 rRNA methyltransferase and ribosome maturation factor (BUD23/WBSCR22) and 28S rRNA Cytosine-C5-methyltransferase (NSUN5A/WBSCR20) were deleted in Williams-Beuren syndrome [20]. Mutations in NSUN7 resulted in sperm motility defects and infertility [232, 359, 360].

#### A-to-I editing dysregulation in genetic and developmental disorders

ADAR1 mutations caused the autoimmune disorder Aicardi-Goutières syndrome (AGS), which was associated with increased expression of interferon-stimulated genes, suggesting that ADAR1 may be a suppressor of Type I interferon signaling [361, 362]. Patients with AGS-causing ADAR1 mutations tended to have aberrant interferon expression and immune responses, which could be rescued by restoring the expression of editing-active cytoplasmic ADARs [363]. Piana et al. reported bilateral striatal necrosis shown in brain MRI and CT scans of two patients with a clinical diagnosis of AGS caused by ADAR1 mutations [364]. The crystal structures of human ADAR2 deaminase domain revealed that AGS-causing mutations might influence RNA binding and catalysis through three types of mutations, including mutations on RNA-binding loops, mutations that alter RNA-binding loop disposition, and mutations that change the position of an α-helix bearing an essential catalytic residue [365].

Dysregulation of the IFN-inducible p150 ADAR1 isoform led to embryonic lethality at E11-E12 [366]. The p150-deficient mouse embryo fibroblasts (MEFs) showed extensive syncytium formation and cytopathic effect after Measles viral infection, which may lead to subacute sclerosing panencephalitis if persistently infecting the central nervous system [366]. Mice with altered 5HT2CR RNA adenosine-to-inosine editing displayed characteristics of Prader-Willi Syndrome, including decreased somatic growth, failure to thrive, neonatal muscular hypotonia, and reduced food consumption followed by post-weaning hyperphagia [367]. 

#### Dysregulation of other RNA modifications in genetic and developmental disorders

##### m6A

The 18S rRNA can be m6A methylated at position A1832 by METTL5, absence of which resulted in decreased global translation rate, compromised differentiation potential, and spontaneous loss of pluripotency in mouse ESCs. Mice deficient in METTL5 were born at non-Mendelian rates and developed morphological and behavioral abnormalities, recapitulating symptoms of patients with DNA variants in METTL5 [145]. Contradictory results have been reported, where in some research, m6A modification destabilized developmental regulators to maintain self-renewal and pluripotency of ESCs [48, 368], while other studies emphasized the requirement of m6A for cell fate transition of ESCs to differentiated lineages rather than ESC maintenance [48].

##### m7G

WDR4 mutation impaired m7G46 methylation of specific tRNA species and caused a distinct form of microcephalic primordial dwarfism [369] and was likely the cause for Galloway-Mowat syndrome in an Indian family, who displayed phenotypes such as developmental delay, growth deficiency, intellectual disability, and microcephaly [370]. Mettl1 knockout mouse ESCs showed increased ribosome occupancy at the corresponding codons and impaired translation of cell cycle genes and those associated with brain abnormalities [371]. Moreover, Mettl1 or Wdr4 knockout mouse ESCs displayed defective self-renewal and neural differentiation, highlighting the essential role of Mettl1/Wdr4-mediated m7G tRNA methylome in ESCs [371]. METTL1-mediated m7G modification also played a critical role in the regulation of human induced pluripotent stem cells (hiPSCs) pluripotency and differentiation, as well as in vascular development and vascular disease treatment [372].

##### Ψ

DKC1, a highly conserved orthologue of rat NAP57 and *Saccharomyces cerevisiae* CBF5, is the gene responsible for X-linked dyskeratosis congenital [242]. In 2009, Armistead et al. reported that D86G mutation in the 18S ribosome assembly protein EMG1 N1-specific Ψ methyltransferase was possibly the cause of Bowen-Conradi syndrome [373].

##### Ac4C

NAT10 affected nuclear architecture in human laminopathies, including the premature-aging disease Hutchinson-Gilford progeria syndrome [21]. NAT10-mediated ac4C acetylation of runt-related transcription factor 2 (RUNX2) mRNA spurred osteogenesis of bone marrow-derived mesenchymal stem cells (BMSCs) and prevented ovariectomy-induced bone loss [374]. NAT10-mediated ac4C modification was also required for important functions during meiosis in male germ cells, such as homologous chromosome synapsis, meiotic recombination and repair of DNA double-strand breaks [375]. Loss-of-function variants in THUMPD1 resulted in a loss of ac4C modification in small RNAs and of individually purified tRNA-Ser-CGA, leading to a syndromic form of intellectual disability associated with behavioral abnormalities, developmental delay, facial dysmorphism, and hearing loss [376].

#### Summary of RNA modification dysregulation in genetic and developmental disorders

Human embryonic development is a highly complex process. Our knowledge of the full spectrum of RNA modifications in the context of genetic and developmental disorders is still limited. Genetic and developmental disorders are characterized by genetic heterogeneity, meaning that different mutations in various genes may result in similar phenotypes. This genetic heterogeneity often leads to contradictory findings as studies focus on specific gene mutations or patient populations. Investigating RNA modifications that are dysregulated in specific genetic and developmental disorders, such as autism spectrum disorders, intellectual disabilities, or congenital anomalies, will certainly provide valuable insights into their role in disease pathogenesis. Identifying disease-specific RNA modification patterns or signatures may lead to the development of diagnostic markers and potential targets for therapeutic intervention. Examining the dynamic changes of RNA modifications during different stages of development will also provide insights into their functional roles and potential as regulatory mechanisms. Investigating how modifications are established, maintained, and remodeled during embryogenesis and organogenesis will shed light on their significance in development and disease. While the field of RNA modifications holds promise for potential therapeutic interventions and diagnostic markers, translating this knowledge into clinical applications for genetic and developmental disorders is a challenge. Further research is needed to explore the therapeutic potential of targeting RNA modifications and to develop effective strategies for modulating RNA modifications in a precise and controlled manner.

### Immune disorders

It is becoming evident that many viral RNAs are often modified by human RNA modifying enzymes following infection, which may affect their own translation and subsequent viral production, raising an intriguing possibility of targeting these pathways as anti-viral therapies [165].

Flavivirus Zika (ZIKV) RNA was richly methylated with m6A, which could be regulated by host methyltransferases METTL3/METTL14 and demethylases ALKBH5/FTO, ultimately affecting ZIKV production [377]. The m6A readers, YTHDF family proteins, bound to ZIKV RNA, suppressing ZIKV replication [377]. On the other hand, ZIKV infection also altered m6A location in host mRNAs, methylation motifs, as well as target genes modified by methyltransferases [377]. m6A in Flaviviridae viral RNA genomes could be modulated by host m6A-related enzymes, thereby influencing infectious particle production [99]. Recruitment of the cellular YTHDF m6A “reader" proteins to 3' UTR m6A sites in HIV-1 mRNAs strongly enhanced viral gene expression [100]. By studying the viral-host RNA methylomes during HIV-1 infection of human T cells, Lichinchi et al.showed that viral infection triggered a massive m6A increase in both host and viral mRNAs [378]. They identified 14 methylation peaks in HIV-1 mRNA as well as 56 human gene transcripts that were possibly involved in functions in viral gene expression [378]. They also found that methylation of two conserved adenosines in the HIV-1 Rev response element (RRE) RNA enhanced in vivo recruitment of HIV-1 Rev protein to RRE and influenced RNA nuclear export [378].

NAT10-mediated ac4C modification boosted human HIV-1 gene expression by increasing viral RNA stability [379]. NAT10 also regulated neutrophil pyroptosis in sepsis via acetylating UNC-52-like kinase 1 (ULK1) RNA, loss of which activated STING-IRF3-NLRP3 axis signaling pathway [380].

Our understanding of the roles of RNA modifications in immune disorders is very limited. Comprehensive profiling and characterization of RNA modifications in immune cells and tissues are definitely needed to uncover their roles and functional significance in immune disorders. Examining the dynamic changes of RNA modifications during immune responses, in different immune cell subsets, or in the presence of immune stimuli can also help us understand their functional roles. The potential of RNA modifications as therapeutic targets or diagnostic markers for immune disorders holds promise. However, much further research is still needed to determine the therapeutic potential of targeting RNA modifications in immune disorders and to develop effective strategies for modulating RNA modifications in immune cells.

## Targeting RNA modifications for therapeutic purposes

Currently, many research groups in the RNA modification field have been putting effort in developing therapies targeting RNA modification or related enzymes. Several small molecule inhibitors against certain RNA modifying enzymes have been developed and are being tested as potential therapeutics.

### Targeting RNA methylation

We have discussed the important roles RNA methylation, including m6A, m5C, m1A and m7G, played in the malignant biological behavior of tumors as well as other common disease models. Developing specific inhibitors against RNA methylation related proteins is of great clinical value [381].

#### Inhibitors against the METTL family

**Fig. 5:**
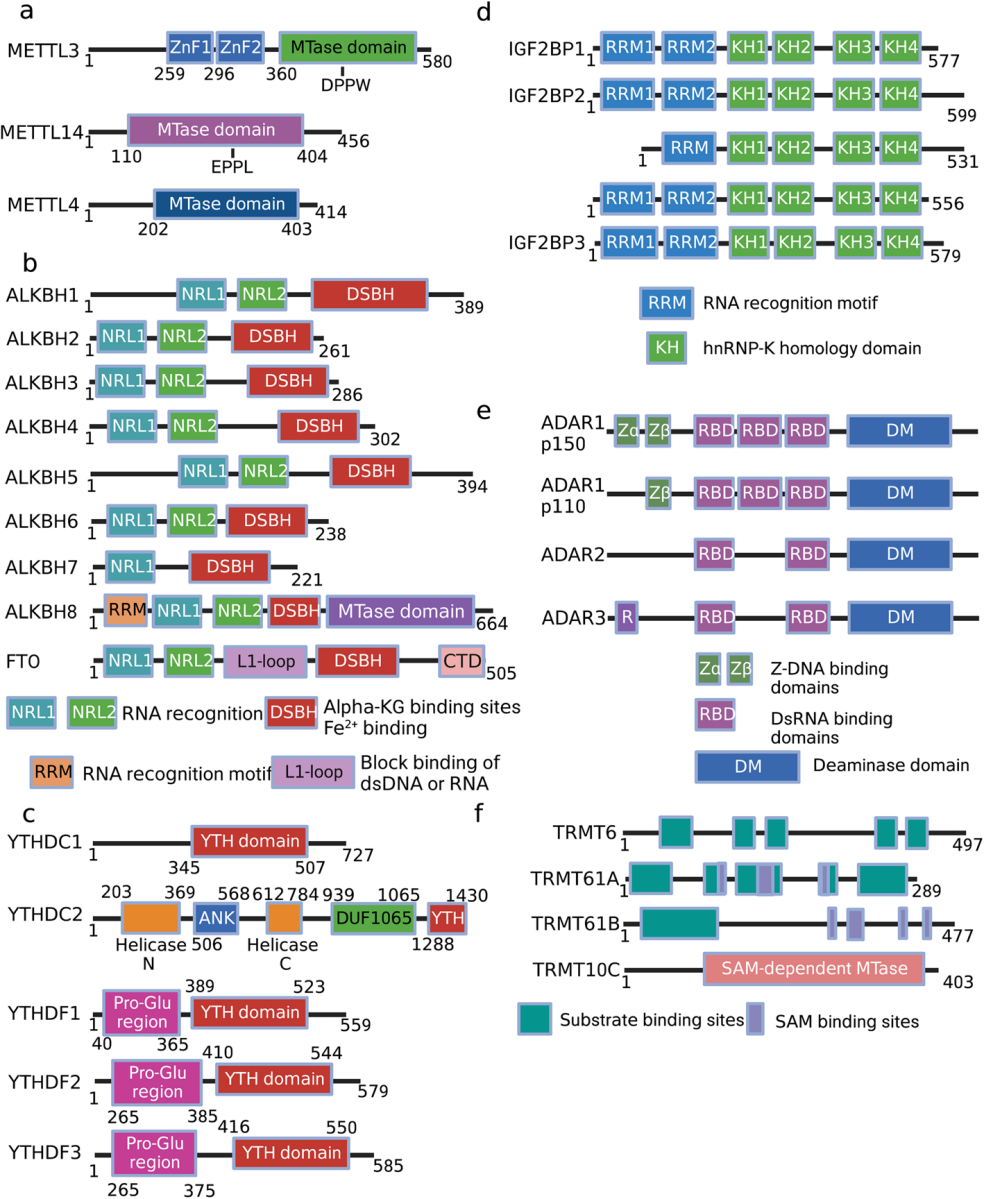
The domain structures of RNA modifying enzyme families. **a** METTL family members each contains a MTase domain that catalyzes the methylation. **b** ALKBH family members and FTO all contain Fe^2+^ binding and α-KG binding sites. **c** YTH-domain-containing family consists of YTHDC1/2 and YTHDF1/2/3. **d** IGF2BP family members each contains four KH domains and at least one RRM domain. **e** ADAR family binds dsRNA through RBD domains, whereas DM domains function as deaminase. **f** TRMT6 only possesses substrate binding sites, whereas TRMT61A/B contains substrate binding sites as well as SAM binding sites. TRMT10C functions as methyltransferase through its SAM-dependent MTase domain. The domain information of TRMT family proteins comes from the Uniprot database

The METTL (methyltransferase-like) proteins are a family of methyltransferases responsible for catalyzing the transfer of a methyl group from a donor molecule (such as S-adenosyl methionine) to specific target sites on RNA molecules [382]. The vertebrate METTL family consists of 33 members, including METTL1, METTL3, METTL5, METTL6, METTL14, and METTL16, each possesses a conserved SAM-binding domain residing in part of the overall 7BS structure (Fig. 5a) [383]. Although enzymes modifying similar substrates did not share a common ancestor, they do largely group together phylogenetically (Fig. 1). The primary function of METTL proteins is to add methyl groups to specific nucleotides within substrate molecules, including proteins, nucleic acids, and other small molecule metabolites. Fourteen of the 33 METTL family members methylate DNA or RNA [383]. The most extensively studied RNA methylation target of METTL proteins is m6A, catalyzed by the METTL3-METTL14 m6A methyltransferase complex. Accumulating evidence has shown that METTL3 may act as a potential therapeutic target, dependent or independent of m6A modification [384]. Most small molecule inhibitors targeting the METTL family have been developed against METTL3.

**Fig. 6:**
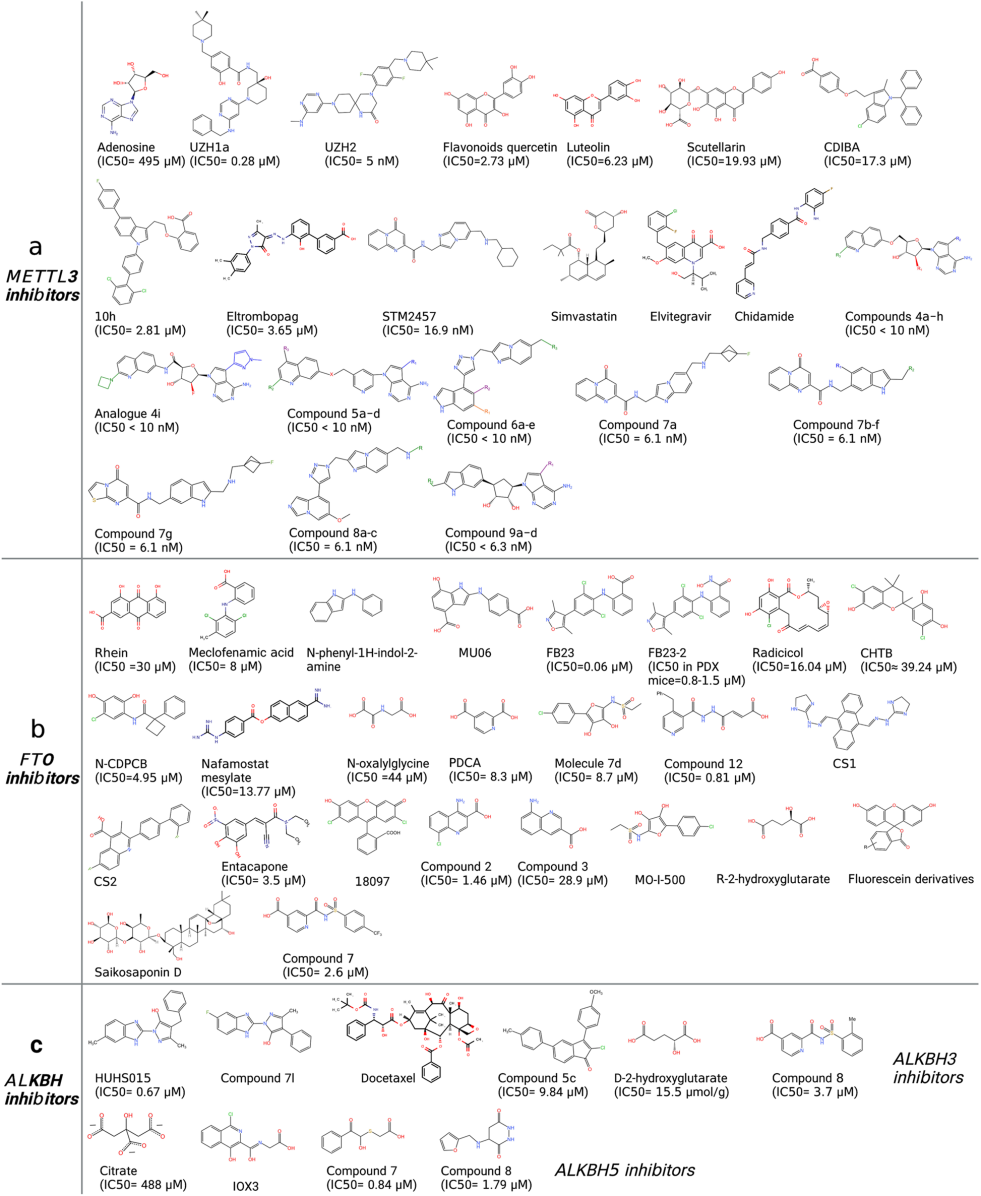
The chemical structures of inhibitors targeting RNA methylation. **a** Inhibitors against METTL3, **b** Inhibitors against FTO and **c** Inhibitors against ALKBH 3/5

Given the continually expanding roles of METTL3 reported in various pathologies, the development of METTL3 inhibitors attracts increasing attention in the research field. Nevertheless, the journey of developing METTL3 inhibitors started quite recently, mostly regarding the MTD of METTL3 as the main target for inhibitor design [385]. Adenosine was the first reported METTL3 inhibitor (IC50 = 495 μM) that acted with a SAM-competitive mode of action, since it overlapped with the adenosine portion of both SAM and the product S-adenosylhomocysteine (SAH, Fig. 6a) [385]. Bedi et al.screened a library of 4000 adenosine analogue and derivatives by high-throughput docking into METTL3 and identified seven compounds that showed good ligand efficiency, adenosine analogues showed poor cellular permeability properties as well as poor selectivity against other methyltransferases. Therefore, research continued seeking non-nucleoside selective METTL3 inhibitors, leading to the discovery of the high-nanomolar inhibitor UZH1a [386]. UZH1a was developed through a structure-based optimization approach along with potency evaluation of compounds in a homogeneous time-resolved fluorescence (HTRF) enzyme assay (IC50 = 0.28 μM) (Fig. 6a) [387]. UZH1a was selective over a panel of other SAM-dependent methyltransferases and a panel of kinases, and was highly permeable in a panel of cell lines, including colorectal adenocarcinoma epithelial cells Caco-2 [385, 388]. Other than its activity in colorectal cancer cells, UZH1a was also shown to decrease m6A methylation in multiple cell lines, including the human bone osteosarcoma epithelial cell line U2OS, the AML cell line MOLM-13, and the immortalized human embryonic kidney celine HEK293T. [385, 386] The Caflisch group further optimized the structure of UZH1a, starting from the compound JMC-1 (IC50 = 7 μM) to eventually obtain a 2,5-difluoro analogue UZH2, which was the first single-digit nanomolar METTL3 inhibitor (IC50 = 5 nM) and was highly cell-permeable, although with lower metabolic stability (Fig. 6a) [385, 389]. UZH2 was able to reduce the m6A level of polyadenylated RNA in the AML cell line MOLM-13 and the prostate cancer PC-3 cells [389]. Additionally, UZH2 selectively targeted METTL3 over other RNA methyltransferases, such as METTL16 and METTL1 [388].

Natural products, flavonoids quercetin, luteolin and scutellarin, have recently been identified as METTL3 inhibitors, with IC50 values of 2.73, 6.23, and 19.93 μM, respectively (Fig. 6a). Flavonoids quercetin decreased the m6A level and impaired cell viability in the human pancreatic adenocarcinoma MIA PaCa-2 cells as well as the hepatocellular carcinoma Huh7 cells [385]. Notably, polyphenolic compounds are known to have pleiotropic activity. For example, luteolin has been shown to also modulate multiple epigenetic enzymes, including HDAC1, DNMT1, SIRT6 and p300, as well as topoisomerases I and II [390]. Therefore, these compounds may only be considered as starting points for developing new optimized derivatives [385].

*CDIBA* was the first METTL3 allosteric inhibitor (IC50 = 17.3 μM) reported in the literature, identified through a screening of a Korea Chemical Bank compound library (Fig. 6a) [391]. CDIBA was initially reported as a cytosolic phospholipase A2 (cPLA2) inhibitor [392]. Optimization of CDIBA aiming to improve the METTL3 inhibitory activity led to a series of CDIBA derivatives with better potency. The most potent compound among these derivatives was 10 h (IC50 = 2.81 μM), which was shown to inhibit cell proliferation of AML cell lines THP-1, HL60, and MOLM-14, as well as to suppress m6A levels in MOLM-13 cells (Fig. 6a) [385].

A known thrombopoietin receptor agonist, eltrombopag, has recently been reported as a potential allosteric inhibitor of the METTL3/14 complex (IC50 = 3.65 μM) (Fig. 6a) [393–395]. Eltrombopag displayed high selectivity for METTL3-METTL14 over five histone methyltransferases (SETD2, G9a, DOT1L, PRMT1, and SMYD3), with a slight cross-influence on the MLL4 complex and PRDM9 [385]. Eltrombopag was also tested in MOLM-13 cells, where it inhibited cell proliferation and reduced the m6A levels in a dose-dependent manner. Moreover, eltrombopag displayed synergistic antiproliferative activity in combination with the approved AML drug venetoclax, a BCL-2 inhibitor, when tested in MOLM-13 cells [385, 396].

Through a high-throughput screening of 250,000 compounds, Kouzarides’ team identified STM1760 (IC50 = 51.7 μM), structural optimization of which led to STM2457 (IC50 = 16.9 nM) (Fig. 6a) [385, 397]. STM2457 was highly selective for METTL3 over a panel of 45 methyltransferases (including RNA, protein, and DNA methyltransferases) and 468 kinases [397]. STM2457 impaired cell proliferation in a panel of various AML cell lines, induced cell cycle arrest and myeloid differentiation as well as triggered apoptosis. In MOLM-13 cells, STM2457 specifically reduced m6A on mRNA in a dose-dependent manner without affecting other RNA modifications (m^6^Am, m^6^_2_A, and m^7^G) [397]. Analysis through m6A-meRIP-seq coupled with qRT-PCR showed that STM2457 could reduce the amount of m6A on mRNA (and consequent protein level) of METTL3 substrates, such as BRD4, SP1, HOXA10 and MYC, without influencing non-METTL3 mRNA substrates. Daily treatment with 50 mg/kg STM2457 in patient-derived xenograft (PDX) mice blocked the engraftment process and leukemic expansion, extended lifespan of the mice, with fewer human CD45 + cells observed in the spleen and bone marrow and no significant weight loss or toxicity [385, 397].

Simvastatin has been reported to suppress lung cancer cell EMT by downregulating METTL3 and METTL3-mediated EZH2 mRNA m6A modification and protein expression [384, 398]. The integrase inhibitor elvitegravir used currently as anti-HIV treatment, was reported to interact with METTL3 (Fig. 6a) [399]. Liao et al.showed that elvitegravir promoted METTL3 degradation by enhancing its interaction with the E3 ubiquitin ligase STIP1 homology and U-Box containing protein 1 (STUB1), thus inhibiting the invasion capacity of the ESCC cell lines KYSE150-Luc-LM5 and KYSE270. Xenograft mice intravenously injected with KYSE150-Luc-LM5 cells showed a dose-dependent decrease of lung metastasis in the elvitegravir-treated group (at either 5 or 10 mg/kg) [385, 399]. Chidamide, a new small molecule inhibitor targeting HDAC1/2/3/10 [400], was recently revealed to improve NSCLC cell sensitivity to Crizotinib by decreasing the stability and translation of METTL3/WTAP transcripts to decrease the c-Met m6A modification and expression (Fig. 6a) [401].

Fiorentino et al. have also reviewed multiple potential METTL3 inhibitors reported in multiple patents filed by Accent Therapeutics and Storm Therapeutics. Among the compounds designed by Accent Therapeutics, a panel of 2-deoxy-2-fluororibose and 2-deoxyribose derivatives (compounds 4a − h) were the most potent (IC50 < 10 nM) and selective METTL3 inhibitors, among which compound 4a was > 100-fold selective over PRMT5 and METTL1/16 (Fig. 6a) [385]. In addition, compounds 4a − h decreased m6A level in MOLM-13 cellular mRNA and impaired the proliferation. Analogue 4i, a 4a derivative that was also designed by Accent Therapeutics scientists, inhibited METTL3 (IC50 < 10 nM) with an > 100-fold selectivity over FMS-like tyrosine kinase 3 (FLT3) and PRMT5 (Fig. 6a) [385]. Compound 5a − d also exhibited potent METTL3 inhibition (IC50 < 10 nM) and selectivity over METTL1/16 and displayed similar cellular functions in MOLM-13 AML cells, such as decreasing cellular mRNA m6A level and impairing proliferation (Fig. 6a) [385]. METTL3 inhibitors disclosed by Storm Therapeutics scientists, compounds 6a − e (IC50 < 10 nM), compounds 7a-g and 8a-c (IC50 = 6.1 nM), as well as compounds 9a − d (IC50 < 6.3 nM) also inhibited the proliferation of the ovarian adenocarcinoma cell line Caov-3 and the AML cell line MOLM-13 or Kasumi-1 (Fig. 6a) [385].

Although many METTL3 inhibitors have been developed, their specificity and selectivity vary. The challenge in developing highly specific inhibitors lies in targeting METTL3 without affecting other related enzymes or biological processes. Achieving high specificity is crucial to minimize off-target effects and ensure the desired therapeutic outcome. As of our current knowledge, inhibitors against other METTL family proteins have not yet been reported. The METTL protein family is a subject of active research, as scientists continue to explore their functions, target specificities, and the impact of RNA methylation on cellular processes and diseases. Understanding the role of METTL proteins in RNA modification provides insights into the complex regulation of gene expression and the molecular mechanisms underlying various biological processes.

#### Inhibitors against the ALKBH family

The AlkB homolog (ALKBH) family encodes nine homologous enzymes, including ALKBH1-8 and FTO, that demethylate different substrates, including ssDNA, dsDNA, mRNA, tRNA, and proteins depending on Fe2 + and α-KG (Fig. 5b) [402, 403]. All ALKB homologs contain a highly conserved double-stranded β-helix domain (Fe^2+^ binding and α-KG binding domains) [335]. The α-KG binding (ALKB) domain is the only active domain in ALKBH1-7, whereas ALKBH8 has an RNA recognizing motif and an MTase domain and FTO has a C-terminal domain [335]. The different structures of ALKB family members imply their functions [335]. The primary function of eukaryotic ALKBH proteins is to remove alkyl groups from nucleobases, thereby repairing DNA and RNA damage [404]. In addition to their repair function, ALKBH proteins have been found to participate in other biological processes. Particular ALKBH homologs act on a different spectrum of specific substrates. For instance, human ALKBH1 removes the methyl group from lysine on histone H2A and 6 mA from DNA. ALKBH2-3 covers specificity of prokaryotic AlkB3-4, whereas ALKBH5 and FTO remove m6A from mRNA [404].

In line with the important roles FTO demethylase plays in cancer and other diseases, efforts have been made to develop small molecule inhibitors against FTO. The first FTO inhibitor identified, a natural product Rhein (IC50 = 30 μM), inhibited FTO by competitively binding the catalytic domain against ssRNA substrate and increased cellular mRNA m6A levels in a dose-dependent manner (Fig. 6b) [405]. Yan et al. demonstrated that inhibiting FTO demethylase activity with Rhein sensitized resistant cells to tyrosine kinase inhibitors (TKIs) and downregulated B-cell lymphoma-2 (Bcl-2) and MER proto-oncogene, tyrosine kinase (MERTK), suggesting that combinatorial treatment with Rhein and TKIs may be an effective approach to treat leukemia [406]. A recent study by Huang et al.provided further mechanistic insights regarding Rhein modulation of adipogenesis, where the Receptor Expressing-Enhancing Protein 3 (REEP3) was discovered as a m6A-independent target of Rhein [407]. As an endoplasmic reticulum (ER) regulator, REEP3 promoted adipogenesis in an m6A-independent manner and represented a druggable candidate target for obesity therapeutics [407].

The nonsteroidal anti-inflammatory drug, meclofenamic acid (MA), was identified in 2015 as a highly selective FTO inhibitor (IC50 = 8 μM) using a drug repurposing strategy (Fig. 6b) [35]. MA inhibition of FTO led to increased HeLa cell mRNA m6A levels and was independent of 2OG or iron ion chelation [35, 408]. MA is likely bound competitively to FTO instead of its substrate m6A-containing ssDNA [409]. In addition, MA was also reported to either prevent or reverse TKI resistance [406].

Considering the structure and activity of Rhein and MA, a novel scaffold N-phenyl-1H-indol-2-amine was used as a base to design FTO inhibitors [410]. Among all the derivatives, compound MU06 showed the highest FTO binding affinities and was docked into FTO active cavity through H-bond interactions (Fig. 6b) [410].

Based on MA structure, Huang et al.synthesized FB23 (IC50 = 0.06 μM) and FB23-2, which directly bound to FTO and selectively inhibited the m6A demethylase activity of FTO (Fig. 6b) [411]. FB23-2, the derivative of FB23, showed better cell permeability, activity and selectivity, significantly inhibiting proliferation and promoting differentiation/apoptosis of human AML cells and primary blast AML cells in PDX mice (IC50 = 0.8–1.5 μM) compared to FB23 (IC50 = 23.6–44.8 μM) [411].

Chang’s group characterized four FTO inhibitors, including Radicicol [412], CHTB [413] and N-CDPCB [414] and Nafamostat mesylate (NM) [415]. N-(5-Chloro-2,4-dihydroxyphenyl)-1-phenylcyclobutanecarboxamide (N-CDPCB, IC50 = 4.95 μM) bound with FTO at a new site that is not conserved in other mammalian AlkB members, suggesting its potential as a selective inhibitor for FTO (Fig. 6b) [413]. N-CDPCB-treated FTO-overexpressing 3T3-L1 pre-adipocytes showed increased m6A levels in mRNA compared to untreated cells [413]. Similar to N-CDPCB, another FTO inhibitor identified in 2016, 4-chloro-6-(6'-chloro-7'-hydroxy-2',4',4'-trimethyl-chroman-2'-yl)benzene-1,3-diol (CHTB, IC50 ≈ 39.24 μM), bound to FTO at a novel site and completely overlapped with the methylated strand in the dsDNA bound ALKBH2, suggesting that it might act as a competitive FTO inhibitor (Fig. 6b) [414]. Furthermore, many CHTB-interacting residues are not conserved among AlkB members, suggesting that CHTB may be an FTO specific inhibitor [414]. The natural compound Radicicol was identified in 2018 as an effective FTO inhibitor (IC50 = 16.04 μM) that showed dose-dependent inhibition against FTO m6A demethylation activity (Fig. 6b) [412]. Radicicol adopted an L-shaped conformation in the FTO binding site and occupies the same position as N-CDPCB through the conserved 1,3-diol group, although at strikingly different orientations [412]. Nafamostat mesylate (NM), a previously discovered serine protease inhibitor used for the treatment of pancreatitis and cancers, was recently identified as a competitive or allosteric FTO inhibitor (IC50 = 13.77 μM) (Fig. 6b). It's binding to FTO was driven by higher positive entropy changes and smaller negative enthalpy changes [415]. Through comparison, Chang et al. revealed that CHTB had the highest binding affinity for FTO, followed by NM, radicicol and N-CDPCB [415].

The 2OG analogues, N-oxalylglycine (NOG, IC50 = 44 μM) and pyridine-2,4-dicarboxylate (PDCA, IC50 = 8.3 μM), occupied the 2OG binding pocket of FTO by forming electrostatic and hydrogen bond (Fig. 6b) [416]. Screening of other related inhibitors, such as hydroxyquinoline-, pyridyl-, and isoquinoline-based compounds revealed binding across both co-substrate and primary substrate binding sites [416]. To develop novel antiepileptogenic compounds, Zheng et al.also designed a new FTO inhibitor (molecule 7d, IC50 = 8.7 μM) pertaining to the 2OG-dependent enzymatic activity of FTO demethylase (Fig. 6b) [417]. 7d increased cellular RNA m6A methylation in HeLa cells and showed anticonvulsant activity in an animal model of pharmaco-resistant epilepsy [417]. Toh and team identified compound 12 (IC50 = 0.81 μM) as an inhibitor highly selective for FTO over other AlkB subfamilies (including Alkb and ALKBH2/3/5) as well as several other 2OG oxygenases (Fig. 6b) [418].

Through structure based virtual screening, Chen et al. recently found two robust FTO inhibitors CS1 and CS2 with IC50 at nanomolar range in AML cells (Fig. 6b) [419]. CS1 and CS2 selectively occupied FTO catalytic pocket, thus inhibiting FTO demethylase activity by blocking the interaction between m6A-modified substrate and FTO catalytic pocket [419]. Excitingly, CS1 and CS2 were also highly effective in inhibiting AML differentiation and the FTO signaling pathways, as well as in sensitizing AML cells to T cell cytotoxicity by decreasing the expression of leukocyte immunoglobulin-like receptor subfamily B 4 (LILRB4), thus overcoming immune evasion [419].

Using structural and biochemical studies, Peng et al.identified entacapone, an FDA-approved drug for treating Parkinson’s disease [420], as an FTO inhibitor (IC50 = 3.5 μM) that lowered fasting blood glucose concentrations and reduced body weight in diet-induced obese mice (Fig. 6b) [421]. Meanwhile, they also identified the transcription factor forkhead box protein O1 (FOXO1) mRNA as a direct FTO substrate, together modulating gluconeogenesis and thermogenesis in the liver and in adipose tissues in mice, respectively [421].

Through virtual screening, structural optimization and bioassay, Xie et al.developed a novel small-molecule FTO inhibitor, 18,097, which selectively bound to FTO active site and significantly inhibited breast cancer cell proliferation and metastasis both in vitro and in vivo (Fig. 6b) [422]. Meanwhile, 18,097 increased m6A modification abundance on suppressor of cytokine signaling 1 (SOCS1) mRNA, recruiting IGF2BP1 for mRNA stabilization and subsequently activating the P53 signaling pathway (Fig. 3a) [422]. Moreover, 18,097 also attenuated cellular lipogenesis by downregulating peroxisome proliferator-activated receptor gamma (PPARg), CEBPA and CEBPB (Fig. 4a) [422].

Using in silico-based rational target-tailored development methods, Selberg et al. developed two small-molecule FTO inhibitors with potent inhibition, 4-amino-8-chloroquinoline-3-carboxylic acid (compound 2, IC50 = 1.46 µM) and 8-aminoquinoline-3-carboxylic acid (compound 3, IC50 = 28.9 µM), which supported the survival of growth factor-deprived primary dopamine neurons in culture (Fig. 6b) [423]. These FTO inhibitors demonstrated higher potency in protecting DA neurons as compared to the ALKBH5 m6A demethylase inhibitors developed previously by the same group [423].

Another pharmacological FTO inhibitor, MO-I-500, significantly inhibited survival of chemo-resistant SUM149-MA triple-negative inflammatory breast cancer cells and downregulated FTO and IRX3 protein expression in the SUM149 cells initially surviving in glutamine-free medium, whereas it had little effect on cell growth when SUM149 or SUM149-MA cells that were not posed to metabolic challenge (Fig. 6b) [424].

*R-2-hydroxyglutarate (R-2HG)*, originally reported as an oncometabolite highly produced by mutant isocitrate dehydrogenase 1/2 (IDH1/2) enzymes, suppressed leukemia cell proliferation/viability and promoted cell-cycle arrest and apoptosis by inhibiting FTO demethylase activity and manipulating the expression levels of CCAAT/enhancer-binding protein alpha (CEBPA) and MYC (Figs. 3a, 6b) [57]. Treating R-2HG-sensitive leukemic cells with R-2HG markedly increased the m6A methylation of CEBPA/MYC mRNA, leading to increased recognition by YTHDF2 and eventual degradation of CEBPA/MYC transcripts (Fig. 4c) [57].

Wang et al.demonstrated that fluorescein derivatives could serve as bifunctional molecules that simultaneously inhibited and labeled FTO protein [425]. Saikosaponin D exhibited anti-proliferative and apoptosis/cell-cycle arrest promoting activities in AML by targeting the FTO/m6A signaling (Fig. 6b) [426].

The TCA cycle intermediate citrate was a modest ALKHB5 inhibitor (IC50 = 488 μM) (Fig. 6c). Citrate/ALKBH5 co-crystal structure revealed that citrate excluded both Fe(II) and 2OG in ALKBH5 [427]. Moreover, in the study by Aik et al., citrate was also reported as a modest FTO inhibitor (IC50 = 300 μM) [416], where citrate only replaced 2OG but not the Fe(II) site, indicating that citrate was not selective for ALKBH5 or FTO [409]. Studies by Aik and others demonstrated that 2OG oxygenase inhibitors NOG (IC50 = 25.85 μM), PDCA (IC50 = 347.2 μM) and HIF PHD inhibitor IOX3 also showed modest inhibition against ALKBH5 activity by competing with 2OG (Fig. 6c) [428, 429]. Furthermore, ALKBH5 was reported to regulate anti-PD-1 therapy response by modulating lactate content and suppressive immune cell accumulation in tumor microenvironment [46]. A small-molecule inhibitor of ALKBH5 identified through in silico screening, named ALK-04, enhanced the efficacy of anti-PD-1 therapy in melanoma [46]. Selberg’s group also identified two ALKBH5 inhibitors, 2-[(1-hydroxy-2-oxo-2-phenylethyl)sulfanyl]acetic acid (compound 7, IC50 = 0.840 µM) and 4-[(furan-2-yl)methyl]amino-1,2-diazinane-3,6-dione (compound 8, IC50 = 1.79 µM) (Fig. 6c). In growth factor deprivation model, both ALKBH5 inhibitors increased the number of TH-positive neurons. Compound 8 rescued growth factor-deprived dopamine neurons, whereas Compound 7 barely protected from apoptosis induced in E13 dopamine neurons [423].

Nakao et al.designed and synthesized a series of 1-aryl-3,4-substituted-1H-pyrazol-5-ol derivatives as ALKBH3 inhibitors, among which 1-(1H-5-methylbenzimidazol-2-yl)-4-benzyl-3-methyl-1H-pyrazol-5-ol (HUHS015) demonstrated the highest potency (IC50 = 0.67 μM) against ALKBH3 (Fig. 6c). HUHS015 significantly suppressed the growth of hormone-independent DU145 prostate cancer cells in a mouse xenograft model (Fig. 4b) [430]. To improve the bioavailability and anticancer effect of HUHS015, Mabuchi et al.applied HUHS015 sodium salt to increase solubility of the inhibitor [431]. They demonstrated that subcutaneous administration of HUHS015 sodium salt significantly increased the area under the curve 0–24 by eightfold compared to HUHS015 along and increased the suppressive effect on DU145 cell proliferation in a xenograft model [431]. Through a series of stability assays, oral absorbability assays as well as enzymatic and cellular assays, Ueda et al. synthesized a novel potent ALKBH3 inhibitor, compound 7 l, which exhibited more potent inhibitory activities in a xenograft model bearing DU145 tumor (10 mg/kg) compared to that of HUHS015 (32 mg/kg) or docetaxel (2.5 mg/kg), a drug clinically used for androgen-independent prostate cancer (Fig. 6c) [432]. Nigam et al.synthesized, screened and evaluated a panel of arylated indene derivatives as a new class of ALKBH3 inhibitors. Using a robust quantitative assay, they obtained compound 5c (IC50 = 9.84 μM) as an ALKBH3 inhibitor that exhibited modest binding properties and inhibited ALKBH3 function in vitro (Fig. 6c). Treatment with 5c abrogated proliferation of A549 lung cancer cells and enhanced sensitivity to DNA alkylating agent MMS [433]. Through a novel multiprotein dynamic combinatorial chemistry strategy, Das et al.simultaneously identified subfamily-selective probes against two clinically important epigenetic enzymes, FTO (compound 7, IC50 = 2.6 μm) and ALKBH3 (compound 8, IC50 = 3.7 μm) (Fig. 6c) [434].

The oncometabolite D-2-hydroxyglutarate (D-2-HG) inhibited the ALKBH enzymes and sensitized IDH mutant cells to alkylating agents, such as procarbazine and lomustine (CCNU) (Fig. 6c) [435]. Chen et al.further demonstrated that both D- and L-enantiomers of the oncometabolite 2-hydroxyglutarate (2HG) significantly inhibited ALKBH2 and ALKBH3 at pathologically relevant concentrations (73–88% for D-2HG and 31–58% for L-2HG inhibition) [436]. The relative concentration of L-2HG (1.15 μmol/g) was about ten times lower than that of D-2HG (15.5 μmol/g), yet the ALKBH2 and ALKBH3 enzymes were still completely inhibited by L-2HG, possibly due to the higher binding affinity of L-2HG [436]. Bian et al.also showed that copper could inhibit the ALKB family DNA repair enzymes under Wilson's Disease condition, which might be caused by disturbed metabolism of copper ions, whereas it showed no significantly inhibitory effect under normal cellular copper concentrations [437].

Research on ALKB homologs is ongoing, as their protein structures are resolved, their functions have been better understood and corresponding drugs have been designed. Several ALKB homolog inhibitors can regulate iron concentrations and subsequently affect ALKB homolog activity as iron chelators. Although not identical in terms of amino acid sequence, the ALKBH family proteins show similar structures over the active site (Fig. 5b) [438]. However, there is a distinct spatial structure in the NRL motif of ALKBH5 that leaves a large open space over the active site, which may confer substrate selectivity to AlkB family proteins [438]. This structural difference likely determines substrate specificity for the m6A demethylases, FTO and ALKBH5. These structural features certainly provide insight into the mechanisms underlying substrate preference and imply potential strategies for development of selective small-molecule modulators [438].

#### Inhibitors against the YTH-domain containing proteins

The YTH-domain containing proteins are a group of proteins that possess a conserved RNA-binding domain known as the YTH (YT521-B homology) domain, which enables proteins such as YTHDF1-3 and YTHDC1-2 to act as readers of m6A-containing transcripts (Fig. 5c) [439]. Although each YTH family protein seems to function in a different way to influence RNA functions, such as splicing, export, translation and decay, the targets of different readers may overlap in a certain degree [439]. It is not yet well understood how each reader selects its own substrate; possibly reasons may include preferred motifs, phase separation, or the possible assigning function of YTHDF3 and other unknown factors [439].

As discussed earlier, YTH-domain containing proteins have been implicated in various biological processes and disease models. For example, Su et al.demonstrated in the HCC model that YTHDF1 silencing combined with EGFR inhibition synergically suppressed the malignancies of HCC cells, suggesting that YTHDF1 inhibition may be of great value in cancer treatment [440]. Therefore, identification and optimization of inhibitors targeting against the YTH-domain containing proteins may be of significant importance for the development of more effective therapeutic strategies.

**Fig. 7:**
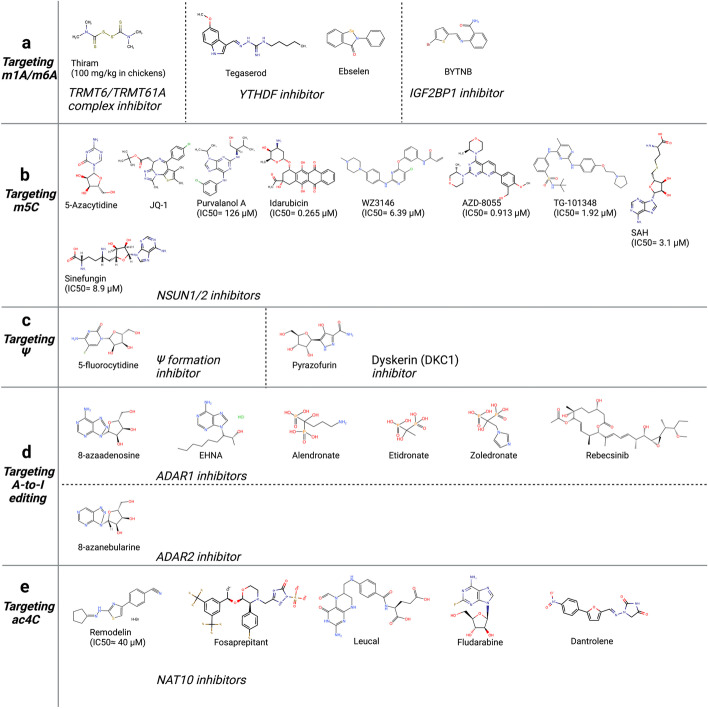
The chemical structures of inhibitors targeting RNA modifications. **a** Inhibitors against m1A/m6A. TRMT6/TRMT61A complex inhibitors target m1A, whereas YTHDF and IGF2BP1 inhibitors target m6A; **b** Inhibitors against m5C; **c** Inhibitors against ψ; **d** Inhibitors against A-to-I editing; **e** Inhibitors against ac4C

Although chemical inhibitors specifically targeting each of the YTH family members are yet to be discovered, investigation of the structural biology of different YTH domains has certainly provided more necessary information for the rational design of small-molecule YTH domain inhibitors [439]. Recently, Micaelli’s group reported a small molecule that bound to YTHDF proteins and interfered with their recognition of m6A-modified RNAs [441]. Through a high-throughput screening aimed at identifying ligands binding in the m6A pocket, they identified the organoselenium compound ebselen as a first-in-class inhibitor of the YTHDF m6A-binding domain (Fig. 7a) [441]. Although ebselen cannot discriminate between the binding domains of the three YTHDF paralogs, this is indeed an important progress in the development of inhibitors against the YTH domain containing proteins [441]. Ebselen's engagement with YTHDF proteins was further validated within cells, interfering with their mRNA binding function [441]. Moreover, they also designed a series of ebselen structural analogs that were able to interact with the YTHDF m6A domain, opening new avenues for the development of disruptors of m6A recognition [441]. In an AML model, Hong et al.identified tegaserod as a potential YTHDF1 inhibitor through a structure-based virtual screening of FDA-approved drugs (Fig. 7a) [442]. Tegaserod blocked YTHDF1 binding with m6A-modified mRNAs, suppressing YTHDF1-mediated translation of Cyclin E2, thus reducing the viability of patient-derived AML cells in vitro, eventually extending survival in patient-derived xenograft models [442].

#### Inhibitors against the IGF2BP family

The insulin-like growth factor-2 mRNA-binding protein (IMP/IGF2BP) family consists of three members, IGF2BP1 (also known as IMP-1 or CRD-BP), IGF2BP2 (IMP-2 or IMP2/Hum2/HuR-BP2), and IGF2BP3 (IMP-3 or KOC) (Fig. 5d) [443, 444]. They are characterized by the presence of multiple RNA recognition motifs (each containing up to six RNA-binding domains) that result in a high complexity of possible modes of interactions with target mRNAs [444]. IGF2BPs primarily bind to the 3'-UTRs of mRNA molecules, although they can also interact with other regions [444].

By binding to specific mRNA targets, IGF2BPs regulate multiple aspects of RNA metabolism, including stability, localization, translation, and alternative splicing, thus influencing several important aspects of cell function, such as cell polarization, morphology, proliferation, migration, metabolism and differentiation [443]. Dysregulation of all three IGF2BPs has been observed in a variety of human cancer types, suggesting their potential as diagnostic or therapeutic targets [444].

Using a fluorescence anisotropy-based assay, Mahapatra et al. screened 160,000 small molecules and identified a potent and selective IGF2BP1 inhibitor, BTYNB, which inhibited melanoma and ovarian cancer cell proliferation by downregulating several IGF2BP1-mediated mRNA transcripts, including c-Myc, β-TrCP1, NF-κB and eEF2 (Fig. 7a) [445]. Therefore, BTYNB may serve as a promising small molecule for further therapeutic evaluation as a treatment for melanoma and ovarian cancer.

#### Inhibitors against the NSUN family and DNMT2

Accumulating evidence has shown that m5C modulates the stability, nuclear export, translation, and cleavage of RNAs to mediate cell differentiation, proliferation, stress responses, apoptosis, and other biological functions [446]. In humans, m5C RNA modification is catalyzed by the NOP2/Sun domain-containing (NSUN) RNA methyltransferase family and DNA methyltransferase 2 (DNMT2) [446]. The NSUN family consists of seven members, including NSUN1-7, each exhibiting different substrate specificities and targeting different RNA molecules [446]. DNMT2 is a member of the DNA methyltransferase family since it was initially identified as a potential DNA methyltransferase due to its sequence similarity to other DNMTs. However, subsequent studies have suggested that DNMT2 primarily functions as an RNA methyltransferase rather than a DNA methyltransferase, specifically modifying tRNA molecules.

Considering the chemical similarity between DNA 5mC and RNA m5C, drugs designed to interfere with DNA methylation may target RNA methylation as well. For example, the RNA m5C methyltransferases DNMT2 and NSUN3 have been suggested to be important targets of the DNA methylation inhibitor 5-Azacytidine (5-AZA) in AML, while the interaction between NSUN1 and the transcriptional co-activator BRD4 induced resistance to 5-AZA (Fig. 7b) [236]. NSUN3 and DNMT2 directly bound heterogeneous nuclear ribonucleoprotein K (hnRNPK), which recruited RNA-polymerase-II through interaction with the lineage-determining transcription factors, CDK9/P-TEFb, GATA1 and SPI1/PU.1, to form a 5-AZA-sensitive chromatin structure (Fig. 4f). On the contrary, NSUN1 interaction with BRD4 and RNA-polymerase-II formed a 5-AZA-insensitive chromatin structure that was yet hypersensitive to the BRD4 inhibitor JQ1 and to siRNA-mediated NSUN1 downregulation (Fig. 7b) [236]. JQ1 and 5-AZA thus showed remarkable synergistic growth inhibition in M2AR leukemia cells [236]. Ma et al.characterized five inhibitors of RNA modification regulators, purvalanol A (IC50 = 126 μM), idarubicin (IC50 = 0.265 μM), WZ3146 (IC50 = 6.39 μM), AZD-8055 (IC50 = 0.913 μM), TG-101348 (IC50 = 1.92 μM), which showed growth inhibitory effect in SH-SY5Y neuroblastoma cells (Fig. 7b). Among these five drugs, WZ3146, AZD-8055 and TG-101348 showed stronger inhibition on NSUN2 expression (Fig. 7b). However, these were not considered as selective inhibitors for NSUN2 since they also had an inhibitory or promotive effect on other RNA modification regulators, including ALYREF, ADAT1, ADAT3, ALKBH5 and TET2 [447]. By using a m5C-sensitive fluorescing probe they developed based on the C3'-endo to C2'-endo sugar-pucker switch structural signatures, Yang et al.determined the IC50 values of two known MTase inhibitors, sinefungin (8.9 μM) and S-adenosyl-L-homocysteine (SAH, 3.1 μM), against NSUN2 in HeLa cells (Fig. 7b) [165, 448–450].

#### Inhibitors against the TRMT family

The TRMTs (tRNA Methyltransferases) are responsible for adding methyl groups to specific nucleotides within tRNA molecules [451–453]. These modifications play important roles in tRNA structure, stability, and function, ultimately influencing protein synthesis and cellular processes. Some well-known TRMTs include TRMT1 [454, 455], TRMT10A [456, 457], TRMT10C [352, 458], TRMT61A [204, 452], TRMT6 [204, 452], and TRMT61B [451, 453], each catalyzing methylation of specific tRNA molecules (Fig. 5f). Disruptions or mutations in TRMT genes have been associated with various human diseases, including cancers, intellectual disabilities, neurological disorders, and mitochondrial diseases. The study of TRMTs provides valuable insights into therapeutically targeting TRMTs.

By screening potential inhibitors blocking TRMT6/TRMT61A interaction from an FDA-approved drug bank, Wang et al.identified a potent inhibitor Thiram against TRMT6/TRMT61A complex that remarkably suppressed self-renewal of liver CSCs and liver tumor growth (Fig. 7a). Thiram in combination with the PPARδ antagonist, GSK3787, synergistically inhibited liver cancer development and tumor growth with high m1A methylations. Meanwhile, Thiram treatment did not affect the levels of other RNA modifications, such as m1G and Ψ [204]. Previous studies showed that oral administration of Thiram (30 μg/day) caused significant inhibition of glioma tumor development and remarkable reduction in metastasis of Lewis lung carcinoma in mice, suggesting that Thiram might be a potential inhibitor of angiogenesis, raising the possibility for its therapeutical use in neovascularization-related pathologies, such as neoplasia [459]. However, high doses of Thiram (100 mg/kg) in chickens could lead to liver damage (Fig. 7a) [204, 460]. Beagle dogs given high doses of Thiram showed severe toxic signs, accompanied by ophthalmological changes, anemia and liver failure, whereas in Wistar rats, although high-dose of Thiram could suppress progression of myocardial lesions of the heart and chronic nephrosis of the kidney as well as decreased mammary fibroadenoma occurrence and skin mass development, it might still lead to anemia, retarded growth, decreased food intake, and regressive changes of the sciatic nerve accompanied by muscle atrophy [461]. Therefore, further preclinical investigation is still needed for therapeutical use of Thiram for cancer treatment, especially in treating HCC patients [204].

### Targeting Ψ

Since Ψ modifications play roles in various cellular processes and diseases, targeting specific PUS family members could therefore potentially affect these processes and have therapeutic benefits.

A partially biotransformed 5-fluorouracil derivative, 5-fluorocytidine, has been reported to specifically inhibit Ψ formation in tRNA (Fig. 7c) [462]. Patton and others have demonstrated that the uridine-to-Ψ conversion in human U1, U2, U4 and U5 small nuclear RNAs was inhibited cognate 5-FU-containing inhibitor snRNAs, raising the possibility for this mechanism to be related to the cytotoxicity of fluoropyrimidines in cancer chemotherapy [463–466]. The incorporation of 5-fluorouracil (5FU) into U2 snRNA blocked pseudouridylation formation in U2 snRNA and pre-mRNA splicing in vivo [467]. Pseudouridine synthase 1 (Pus1p) interacted explicitly with position 27 in the 5FU-containing tRNA anticodon stem without changing the chemical structure of 5FU [468]. However, in 2004, Spedaliere and colleagues showed that not all Ψ synthases were potently inhibited by 5-FU-containing RNAs [469]. They demonstrated in their study that the *Escherichia coli* Ψ synthase TruB containing critical eukaryotic homologs was not inhibited, but rather handled the RNA containing 5-FU as a substrate [469]. The *E. coli* Ψ synthase RluA, on the other hand, was inhibited stoichiometrically by forming a covalent complex with RNA containing 5-FU [469]. Uridine-to-Ψ conversion is reversible, where Ψ can be dephosphorylated by Ψ kinase to Ψ 5'-monophosphate (ΨMP), which is then degraded to uracil and ribose 5-phosphate by ΨMP glycosidase [470, 471]. In 2018, Floresta et al.identified Ψ isoxazolidinyl nucleoside analogues as potential inhibitors of the ΨMP glycosidase [472]. They demonstrated that 5'-monophosphate (isoxazolidinyl derivative 1) could be effectively accommodated within the active site of the enzyme and prevented the first mechanistic step proposed for the degradation of the ΨMP glycosidase, leading to the enzyme inhibition [472]. 5'-monophosphate weakly bound to plasma protein, only moderately permeated the blood–brain barrier, and was non-carcinogenic in rats and mice [472].

Other than 5FU-containing tRNAs, studies have also been conducted searching for potential inhibitors targeting other factors involved in pseudouridinylation. For example, Rocchi et al.sought to identify small molecules able to inhibit the catalytic activity of human dyskerin (DKC1). Using various in silico techniques, they selected compounds and analyzed the binding modes and the interaction patterns of ligands in the human dyskerin catalytic site. They identified four molecules (compound 1, 5, 6, and 10) that significantly inhibited dyskerin pseudouridylation activity, among which only compound 1 (pyrazofurin) showed a significant cytotoxic activity in MCF7 breast cancer cells (Fig. 7c) [473]. Kan et al. later showed that DKC1 depletion or pyrazofurin treatment attenuated colorectal cancer cell proliferation. Combination of pyrazofurin and trametinib, an FDA proved MEK1/2 inhibitor for cancer treatment, synergistically restrained colorectal cancer cell growth in vitro and in vivo [474].

Through screening 270,000 compounds from National Cancer Institute Developmental Therapeutics Program (NCI-DTP) and 4,086 FDA-approved drugs, Cui et al.identified C17 as a potent inhibitor against PUS7 [475]. In cellular tests, C17 strongly suppressed the growth of PBT111, PBT003, PBT726 and PBT707 GSCs (IC50 = 92.15 nM in PBT003 cells) in a PUS7-dependent manner without showing inhibitory effect on control NSCs at 100 nM or even lower concentrations (0–40 nM) [475]. The growth of GSC-derived tumors in NSG mice was also markedly inhibited by C17 treatment, along with decreased Ψ levels, confirming the inhibition of PUS activity by C17 in vivo [475]. Furthermore, the survival of C17-treated NSG mice was dramatically prolonged compared with vehicle-treated mice [475].

Research into Ψ related modifiers as therapeutic targets is still in its early stages, and much more work is needed to fully understand their functions, regulation, and potential for therapeutic intervention. Nonetheless, the emerging understanding of Ψ modifications and their roles in diseases holds promise for future therapeutic strategies.

### Targeting A-to-I editing

ADARs are enzymes catalyzing the chemical conversion of adenosines to inosines in dsRNA substrates [476]. ADAR family enzymes share a common domain architecture consisting of N-terminal dsRNA binding domains (RBDs) and a C-terminal catalytic deaminase domain (DM, Fig. 5e) [476]. Human ADAR1 has a unique domain feature that contains two Z-DNA binding domains that recognize the left-handed helical variant of DNA in a sequence-independent manner (Fig. 5e) [476].

ADAR-mediated A-to-I RNA editing has significant consequences for RNA molecules since inosine is later recognized as guanosine during translation, leading to potential changes in RNA sequences and subsequent protein coding. ADAR-mediated RNA editing is particularly prominent in the nervous system, where it contributes to the diversification of neuronal transcriptomes and fine-tuning of neurotransmitter receptors. ADARs are involved in many physiological and pathological processes, including neuronal development, synaptic plasticity, and neurological disorders. Dysregulation of ADAR activity or RNA editing has been associated with various diseases, including cancer, neurological disorders (such as epilepsy and amyotrophic lateral sclerosis), and autoimmune diseases, suggesting that ADARs may be recognized as potential therapeutic targets. There are ongoing efforts to develop small molecule inhibitors and nucleic acid-based approaches to modulate ADAR activity. These strategies aim to specifically target ADAR enzymes or their binding domains, interfere with RNA editing events, or restore proper RNA editing in disease-associated contexts.

In 2006, Maydanovych synthesized C6-substituted derivatives of 8-azanebularine and showed that C6-methyl derivative was incorporated into an RNA substrate for ADAR2 via the phosphoramidite and showed a strong inhibitory effect on editing enzyme binding, suggesting that methylation at C6 must be considered in the design of new ADAR inhibitors based on this ring system (Fig. 7d) [477]. ADAR1 has been reported as a key factor in Hepatitis B virus (HBV) evasion from IFN responses in hepatocytes, where treatment with the ADAR1 inhibitor 8-azaadenosine significantly enhanced liver immune activation to promote HBV clearance in vivo and in vitro. (Fig. 7d) [478]. ADAR1 up-regulation was observed in gastric cancer and was significantly correlated to metastasis. ADAR1 inhibition with 8-azaadenosine treatment significantly attenuated gastric cancer peritoneal tumor metastasis as well as decreased the expression of the CALR oncogene, the Wnt/β-catenin signaling pathway and EMT process in vivo. Moreover, ADAR1 inhibition also suppressed proliferation and migration of HGC-27 and AGS cells in vitro [479].

Primary cultured cortical cells treated with the ADAR inhibitor erythro-9-(2-hydroxy-3-nonyl) adenine hydrochloride (EHNA) significantly reduced the editing efficacy of 5-HT2CR mRNA in a dose-dependent manner (Fig. 7d) [480].

Through high-throughput screening on 2115 FDA-approved compounds for possible repurposing in inhibition of the Zα domain, Choudhry et al. selected three compounds, alendronate, etidronate, and zoledronate based on their XP Gscore (Fig. 7d) [481]. All three drugs interacted with Lys169/Lys170/Asn173/Tyr177 of ADAR1-like Z-RNA and Z-DNA, whereas only zoledronate showed strong hydrogen bonding and hydrophobic interactions at Arg174, the only differentiating residue between Zα/Z-RNA and Zα/Z-DNA complexes, suggesting its potential as a potent inhibitor against ADAR1 catalytic activity in the A-to-I editing of RNA transcripts [481].

ADAR1p110-to-p150 splice isoform switching induced by inflammatory microenvironment drives cancer stem cell generation and therapeutic resistance in multiple malignancies. Crews et al. developed Rebecsinib, a selective ADAR1 inhibitor that reversed ADAR1 splice isoform switching, thus attenuating LSC self-renewal and extending survival of humanized LSC mice (Fig. 7d) [482].

Failure of GluA2 RNA editing resulting from ADAR2 loss often occurs in ALS cases and causes motor neuron death. Recovering ADAR2 level in the ADAR2-depleted mechanistic sporadic ALS mouse model by intravenously injecting adeno-associated virus serotype 9 (AAV9)-ADAR2 rescued mice from motor neuron death by normalizing TDP-43 expression, suggesting a possibility of using AAV9-mediated ADAR2 gene delivery as a gene therapy for ALS [307].

The development of therapies targeting ADARs is still in its early stages, and it requires a comprehensive understanding of the complex regulatory mechanisms and consequences of RNA editing. Off-target effects and unintended consequences of modulating ADAR activity need to be carefully considered. ADARs have multiple RNA targets, and altering their activity could have broader effects on RNA processing and gene expression. Therefore, while ADARs hold potential as therapeutic targets, further research is needed to fully understand the mechanisms, functions, and consequences of ADAR-mediated RNA editing in various diseases. Overcoming the challenges of achieving target selectivity and minimizing off-target effects will be crucial for the development of effective and safe therapies targeting ADARs.

### Targeting ac4C

The functional significance of Ac4C modifications in RNA is still being actively researched, and their specific roles and implications in cellular processes and disease conditions are not yet fully understood. However, dysregulation of ac4C has been reported in multiple cancer types as well as a few other disease models. Therefore, it is important to screen for inhibitors targeting ac4C modifiers.

The only NAT10 inhibitor reported so far, Remodelin (~ 40 μM), effectively suppressed NAT10 protein expression as well as its activity by binding to its Acetyl-CoA binding site (Fig. 7e) [21, 483]. Romedelin was originally shown to promote chromatin organization, nuclear architecture, and to decrease DNA damage markers in human lamin A/C-depleted cells and Hutchinson-Gilford Progeria Syndrome (HGPS)-derived patient cells [21]. Remodelin treatment led to a notable decline in NAT10 abundance in the cells [21, 484–486]. Due to the important role of NAT10 in cancer progression, Remodelin has proven its anti-cancer therapeutic potential in several cancer types. For example, NAT10 up-regulation promoted HCC cell metastasis through EMT [487]. Treatment with Remodelin led to diminished cell invasion and migration as well as increased E-cadherin decreased vimentin [487]. Remodelin suppresses cancer growth and progression by reducing hypoxia-induced or constitutional expression of HIFs in cells and altering mitochondrial lipid metabolism [488, 489]. NAT10 overexpression was observed in AML patients and was associated with poor outcomes [483]. Targeting NAT10 with Remodelin induced apoptosis by enhancing ER stress in AML cells through the increased expression of G Protein-Coupled Receptor 78 (GRP78) and the cleavage of caspase 12, although at a relatively high dose (125 μM), further supporting the potential of Romedelin in clinical applications [483]. NAT10 promoted multiple myeloma (MM) cell proliferation by catalyzing CEP170 mRNA acetylation to enhance translation efficiency. Treatment with remodeling suppressed MM cellular growth and induced cellular apoptosis in vitro, as well as extended the survival of 5TMM3VT mice in vivo [490]. Remodeling also inhibited HIV-1 replication at levels that showed no inhibitory effect on cell viability, thus identifying NAT10 as a potential target for antiviral drug development [379]. NAT10-catalyzed ac4C acetylation of RUNX2 spurred osteogenesis of bone marrow-derived mesenchymal stem cells (BMSCs) and prevented ovariectomy-induced bone loss. Remodelin treatment enhanced the loss of bone mass in ovariectomized (OVX) mice and decreased RUNX2 mRNA half-life and protein expression in BMSCs, thus attenuating the osteogenic differentiation of BMSCs [374].

Recently, Dalhat screened a library of FDA-approved drugs aiming to identify novel inhibitors of NAT10 activity. They selected four drugs, namely fosaprepitant, leucal, fludarabine and dantrolene that bound to NAT10 with a better binding capability, indicating that these four drugs might serve as potential NAT10 inhibitors, although further cellular testing and evaluation are needed to verify their functions against NAT10 related diseases (Fig. 7e) [491].

At present, the research on ac4C modifications in RNA is still in its early stages, and further investigations are needed to elucidate their functional significance and therapeutic potential. Continued research in this area will contribute to a better understanding of the roles of ac4C in RNA biology and its potential as a therapeutic target.

### Challenges in targeting RNA modifications

Although more and more inhibitors against RNA modifiers have been developed, targeting RNA modifications still face a great challenge.

First, many RNA modifications occur at low abundance, making it challenging to selectively target and manipulate the modified RNA molecules without affecting the unmodified ones. Achieving specificity in targeting a particular modification while minimizing off-target effects is a significant hurdle. RNA modifications may need to be targeted in specific cells or organs. Efficient delivery of therapeutic agents or editing tools specifically to the desired cell types or tissues is also difficult. RNA modifications are often dynamically regulated and vary in response to different physiological or pathological conditions. Targeting and manipulating modifications in real-time or in a specific context may require precise temporal and spatial control, which can be technically demanding. Some RNA modifications may locate in regions of the RNA molecule that are structurally or functionally inaccessible. For example, modifications within highly structured regions or bound by RNA-binding proteins may be difficult to target or modify using traditional approaches.

Second, our knowledge regarding the structures of some RNA modifiers is still quite limited. For example, the domain structures of the NSUN family and the TRMT family members have not been thoroughly reported yet, it is thus more challenging to design inhibitors against these proteins.

Third, the development of effective tools and technologies for targeting and manipulating specific RNA modifications is an ongoing challenge. Techniques such as CRISPR-based systems or small molecules that specifically modulate RNA modifications need to be refined and optimized for different modifications. The functional consequences and biological roles of many RNA modifications are still not fully understood. Targeting specific modifications without comprehensive knowledge of their functional implications may lead to unpredictable side effects or limited therapeutic outcomes. Any therapeutic intervention targeting RNA modifications must consider potential safety concerns and minimize off-target effects.

Addressing these difficulties requires continued research and development of innovative technologies, improved delivery systems, a deeper understanding of modification functions, and rigorous safety assessment. Overcoming these challenges will pave the way for the development of targeted therapies and interventions that leverage the potential of RNA modifications in various biological and disease contexts.

## Conclusion and future perspectives

The study field of RNA modifications is rapidly evolving with ongoing research and discoveries. Despite the discovery of more than 170 different types of RNA modifications, there could still be undiscovered modifications. Identifying novel RNA modifications requires innovative technologies that can accurately detect and characterize these modifications. Mass spectrometry (MS) is a traditional powerful analytical tool for identifying and quantifying RNA modifications. Advanced MS techniques, such as liquid chromatography-mass spectrometry (LC–MS) and RIP-LC–MS, have been instrumental in discovering and characterizing new RNA modifications [1, 492, 493]. Antibody-based methods, such as antibody pull-down or immunoprecipitation, can selectively enrich modified RNA species. For example, antibodies raised against m6A and ac4C have been widely used to study the presence and location of these prevalent RNA modifications. Methylation individual-nucleotide-resolution crosslinking and immunoprecipitation (miCLIP) have been widely used to study RNA methylation [136, 494, 495]. High-throughput sequencing technologies, such as m6A-seq and ac4C-seq have been developed to specifically capture and profile these two RNA modifications, respectively [1, 496]. RiboMeth-seq is a sequencing-based method for mapping and quantifying one of the most abundant RNA modifications, ribose methylation [497]. RiboMeth-seq can also be adapted to other RNA classes, such as mRNA, to reveal new biology involving RNA modifications [498]. DART-seq (deamination adjacent to RNA modification targets) is an antibody-free method for specifically detecting m6A sites. In DART-seq, the cytidine deaminase APOBEC1 is fused to the m6A-binding YTH domain. The fused protein induces C-to-U deamination when expressed in cells at sites adjacent to m6A residues, which can be detected with standard RNA-seq. DART-seq is able to identify thousands of m6A sites in cells from as little as 10 ng of total RNA and can detect m6A accumulation in cells over time [499]. Advancements in artificial intelligence (AI) and machine learning (ML) are revolutionizing the field of RNA modification research. AI and ML algorithms can aid in the analysis of large-scale sequencing data to predict and identify novel RNA modifications by learning patterns and signatures associated with known modifications and applying this knowledge to detect and classify novel modifications. These technologies, along with advancements in data analysis and bioinformatics tools, have significantly contributed to the identification and characterization of known RNA modifications. However, challenges still exist in accurately mapping the exact locations and frequencies of certain modifications at single-nucleotide resolution. Continued development and refinement of advanced techniques and tools for high-throughput detection, mapping, and quantification of RNA modifications is an active area of research. These include innovative sequencing approaches, chemical and immunological enrichment methods, and computational algorithms for data analysis. Such advancements will enable comprehensive profiling of RNA modifications in different cellular contexts and facilitate the discovery of novel modification sites. Additionally, it is important to combine multiple complementary technologies and approaches to achieve comprehensive and accurate characterization of RNA modifications.

It is important to note that there are differences in the identification of RNA modifications among different RNA species such as mRNA, tRNA, rRNA, and ncRNA. These differences arise due to variations in the abundance, structure, modification patterns, and techniques employed for each RNA species. mRNA is generally more abundant compared to tRNA, rRNA, and ncRNA, making it easier to obtain sufficient quantities for analyses. Considering these differences, it is important to tailor the experimental approaches and techniques for the identification and analysis of RNA modifications based on the specific RNA species of interest. Researchers often employ a combination of techniques and methodologies to comprehensively study modifications across different RNA types and unravel their functional significance.

RNA modifications are increasingly recognized for their roles in human diseases, including cancer, neurological disorders, cardiovascular diseases, metabolic disorders and genetic diseases. However, there is still much to learn about the functional consequences of RNA modifications in disease contexts and their potential as therapeutic targets. So far, only a few RNA modifications have been well-characterized, and a great majority of the 170 currently discovered RNA modification types are still poorly understood regarding their biological functions, not to mention their clinical relevance and therapeutic potential. The enzymes responsible for RNA modification are not yet well characterized except for m6A, much work is needed to fully understand the specific mechanisms by which they catalyze the modification of RNAs. Understanding the functional consequences and regulatory mechanisms of RNA modifications is crucial for deciphering their roles in cellular processes. This includes investigating how RNA modifications influence RNA structure, stability, localization, translation, and interactions with other molecules, as well as elucidating the enzymes responsible for modifying RNA and their regulation. Researchers are actively exploring novel modifications, such as ac4C, m1A and m6Am, to understand their functional roles, regulatory mechanisms, and implications in various biological processes, including development, disease, and epigenetic regulation.

It is necessary to mention that RNA modifications do not function in isolation but often interact with and influence each other. Investigating the interplay and cross-talk between different RNA modifications will provide insights into their synergistic effects, functional consequences, and potential regulatory networks. Further investigations into the epitranscriptomic landscape of disease samples, such as patient tissues or biofluids, may lead to the identification of disease-specific RNA modifications, potential diagnostic markers, and therapeutic targets. Addressing these gaps will advance our understanding of RNA modifications and their significance in biological processes and disease pathogenesis and pave the way for the development of novel therapies and diagnostic tools targeting RNA modifications.

With the growing appreciation of the functional importance of RNA modifications, there is interest in developing targeted editing technologies to precisely modulate specific RNA modifications. Such technologies, including base editors or chemical modification tools, can be used to investigate the functional consequences of specific modifications and potentially correct disease-associated dysregulation. CRISPR-Cas systems have also been adapted to develop RNA-targeting tools, such as RNA-targeting Cas proteins and RNA-targeting guide RNAs, which can be used to specifically enrich or manipulate RNA modifications for downstream analysis. For instance, the RNA-targeting Cas13 enzyme has been utilized for the detection and characterization of RNA modifications [500]. Targeting RNA modulators with small molecule inhibitors have also shown promising therapeutic benefit in cancer as well as in other disease models. RNA-modification-based therapies are a promising approach for treating a wide range of diseases, but their safety and efficacy have been a concern for many researchers and healthcare providers. Several strategies have been applied by researchers in the development of inhibitors targeting these RNA modifiers, including identification of natural products, chemical improvement of naturally available compounds, and re-purposing of FDA-approved drugs. Although cellular evaluation has been done for many of these inhibitors, further validation in cellular and animal models is still necessary for some of the compounds to clearly demonstrate their therapeutic efficacy, clinical trials are definitely needed for many of the small molecules discussed in the review. A main concern of targeting RNA modifications for therapeutic purposes is the potential unwanted side effects due to the extensive involvement of each RNA modification in multiple biological pathways.

Taken together, RNA modifiers can be promising targets for clinical therapies. Targeting RNA modifiers may potentially transform the way we diagnose, treat, and prevent a variety of diseases. RNA modification has been shown to play a role in the development and progression of various types of cancer, neurological disorders, cardiovascular diseases, metabolic disorders and genetic disorders. Targeting RNA modification has the potential to be an effective therapeutic strategy for the treatment of these human disease by modulating RNA stability and translation of important proteins. Continued research in the field of RNA modification can certainly improve our understanding of the biological roles of RNA modifications, improve existing therapies, as well as develop new therapies. As our understanding of RNA modification continues to deepen, it may lead to new breakthroughs in the diagnosis, treatment, and prevention of human diseases.

## Data Availability

Not applicable.
